# A highly multiplexed droplet digital PCR assay to measure the intact HIV-1 proviral reservoir

**DOI:** 10.1016/j.xcrm.2021.100243

**Published:** 2021-04-12

**Authors:** Claire N. Levy, Sean M. Hughes, Pavitra Roychoudhury, Daniel B. Reeves, Chelsea Amstuz, Haiying Zhu, Meei-Li Huang, Yulun Wei, Marta E. Bull, Noah A.J. Cassidy, Jan McClure, Lisa M. Frenkel, Mars Stone, Sonia Bakkour, Elizabeth R. Wonderlich, Michael P. Busch, Steven G. Deeks, Joshua T. Schiffer, Robert W. Coombs, Dara A. Lehman, Keith R. Jerome, Florian Hladik

**Affiliations:** 1 Department of Obstetrics & Gynecology, University of Washington, Seattle, WA, USA; 2 Vaccine and Infectious Disease Division, Fred Hutchinson Cancer Research Center, Seattle, WA, USA; 3 Department of Laboratory Medicine and Pathology, University of Washington, Seattle, WA, USA; 4 Seattle Children’s Research Institute, Seattle, WA, USA; 5 Department of Pediatrics, University of Washington, Seattle, WA, USA; 6 Human Biology Division, Fred Hutchinson Cancer Research Center, Seattle, WA, USA; 7 Department of Medicine, University of Washington, Seattle, WA, USA; 8 Department of Global Health, University of Washington, Seattle, WA, USA; 9 Vitalent Research Institute, San Francisco, CA, USA; 10 Department of Laboratory Medicine, University of San Francisco, San Francisco, CA, USA; 11 School of Medicine, University of San Francisco, San Francisco, CA, USA; 12 Department of Infectious Disease Research, Southern Research, 431 Aviation Way, Frederick, MD, USA; 13 Division of HIV, Infectious Diseases and Global Medicine, Zuckerberg San Francisco General Hospital, San Francisco, CA, USA; 14 Clinical Research Division, Fred Hutchinson Cancer Research Center, Seattle, WA, USA

**Keywords:** HIV cure, HIV reservoir, digital PCR, multiplexing, viral outgrowth assay, intact proviral DNA assay, IPDA, genital mucosa, intestinal mucosa, rectal

## Abstract

Quantifying the replication-competent HIV reservoir is essential for evaluating curative strategies. Viral outgrowth assays (VOAs) underestimate the reservoir because they fail to induce all replication-competent proviruses. Single- or double-region HIV DNA assays overestimate it because they fail to exclude many defective proviruses. We designed two triplex droplet digital PCR assays, each with 2 unique targets and 1 in common, and normalize the results to PCR-based T cell counts. Both HIV assays are specific, sensitive, and reproducible. Together, they estimate the number of proviruses containing all five primer-probe regions. Our 5-target results are on average 12.1-fold higher than and correlate with paired quantitative VOA (Spearman's ρ = 0.48) but estimate a markedly smaller reservoir than previous DNA assays. In patients on antiretroviral therapy, decay rates in blood CD4^+^ T cells are faster for intact than for defective proviruses, and intact provirus frequencies are similar in mucosal and circulating T cells.

## Highlights

HIV cure studies rely on precise HIV reservoir assays that can be done at scale
Multiplexed droplet digital PCR can probe multiple HIV genome targets per sample
HIV-1 proviruses containing 5 probed genomic regions are likely intact
PCR-based counting of T cells improves reservoir quantification in tissues

## Graphical abstract

**Figure:**
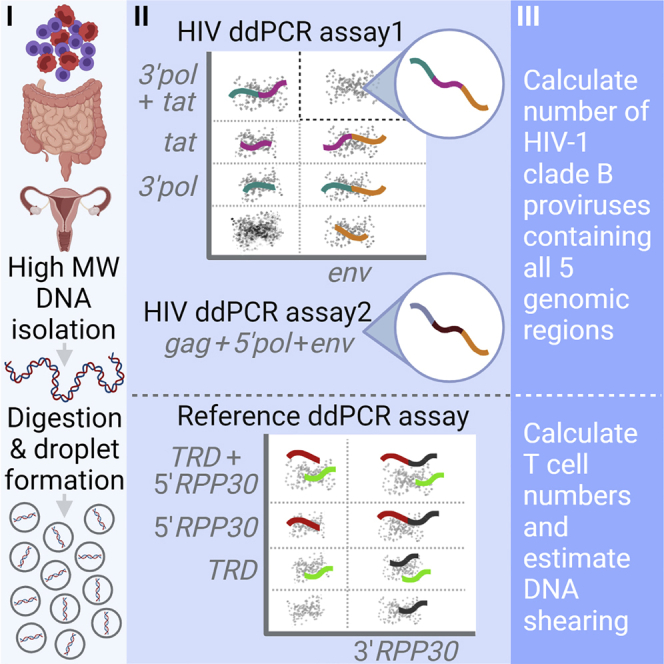

## Teaser

Evaluation of HIV cure interventions requires accurate and scalable measurement of the replication-competent HIV reservoir. Levy at al. describe a highly multiplexed droplet digital PCR assay that simultaneously quantifies likely intact HIV-1 proviruses and T lymphocytes. They validate the assay in cell and tissue specimens from several patient cohorts.

## Introduction

HIV cure studies require a precise method to quantify replication-competent HIV provirus, the major barrier to a cure. A clinically relevant reservoir assay requires high sensitivity and specificity, a relatively small amount of blood or tissue, and a short turnaround time. Ideally, it should also include quantification of the number of potential HIV target cells, because the proportion of latently HIV-infected cells to total relevant target cells can vary greatly between different specimen types, such as blood and mucosal tissues. Quantitative viral outgrowth assays (QVOAs) have been the gold standard for measuring the replication-competent reservoir. However, QVOAs are labor intensive, take days to weeks to culture virus *in vitro* (even with adapted shortened protocols),^1^, ^2^, ^3^ and require a significant amount of blood (∼200 mL). In addition, QVOA fails to stimulate all intact proviruses to replicate^4^ and therefore underestimates the size of the intact reservoir.

PCR-based assays do not rely on cell culture, are highly sensitive, and require less blood volume than QVOAs. However, the utility of a PCR assay strongly depends on the chosen genomic target(s). In the case of HIV-1, quantifying the number of HIV DNA copies using a single conserved target PCR assay greatly overestimates the size of the replication-competent reservoir because most integrated proviruses are defective.^4^, ^5^, ^6^, ^7^, ^8^, ^9^, ^10^, ^11^, ^12^ A single target assay cannot distinguish intact proviruses from those with deletions and/or loss-of-function mutations.^5^, ^6^, ^7^ Reliable estimates of genetically intact proviruses require verification that multiple regions of the HIV genome are present *and* that the detected target sequences are from the same proviruses.

Sequencing full-length proviral clones derived from limiting dilutions can achieve accurate reservoir quantification, but many dozens of sequencing reactions with replicate wells from each patient must be performed to estimate the number of replication-competent proviruses,^13^^,^^14^ the relevant endpoint for HIV eradication protocols. This is prohibitively laborious and expensive for most clinical situations. The emergence of droplet digital PCR (ddPCR) technology offers an alternative.^15^ During ddPCR, PCR reactions, including the input template DNA, are partitioned into thousands of individual droplets and PCR results for each droplet are reported separately. Because HIV-infected cells in antiretroviral treatment (ART)-treated patients are rare (≤1–1,000/million T cells), DNA extracted from patient samples can be added to the ddPCR reaction such that each droplet typically contains human genomic DNA, and either 0 or 1 HIV provirus, with multiple proviruses rarely if ever found in a single droplet.^16^ Therefore, if several regions of proviral DNA are simultaneously targeted in each individual droplet in a multiplexed ddPCR, the number of intact proviruses, i.e., those containing all primer/probe regions, can be accurately estimated.

A ddPCR assay protocol reported in 2019 made use of such multiplexing, probing two regions of the HIV-1 genome within each droplet.^17^ We use two 3-region (triplex) ddPCR assays to develop a 5-region test (1 overlapping region allows inter-assay quality control). We call triple-positive ddPCR droplets “potentially intact.” By combining the two parallel triplex assays, we confidently quantify truly intact HIV-1 viral genomes. As a further enhancement, we adapted a multiplexed ddPCR assay specifically quantifying T cells to accurately normalize to the number of HIV target cells interrogated.^18^ This additional step is especially useful for tissue biopsies, because, in contrast to blood, cell populations in tissues are difficult to isolate and purify.

After validation, we apply our assay to longitudinal blood and mucosal samples from HIV-1-infected patients on ART. We assess the intact reservoir size measured by our assay and directly compare it to QVOA results and *in silico* to a provirus sequence database.

## Results

### Assay development

#### Design of the two HIV-1 triplex assays

**Figure 1:**
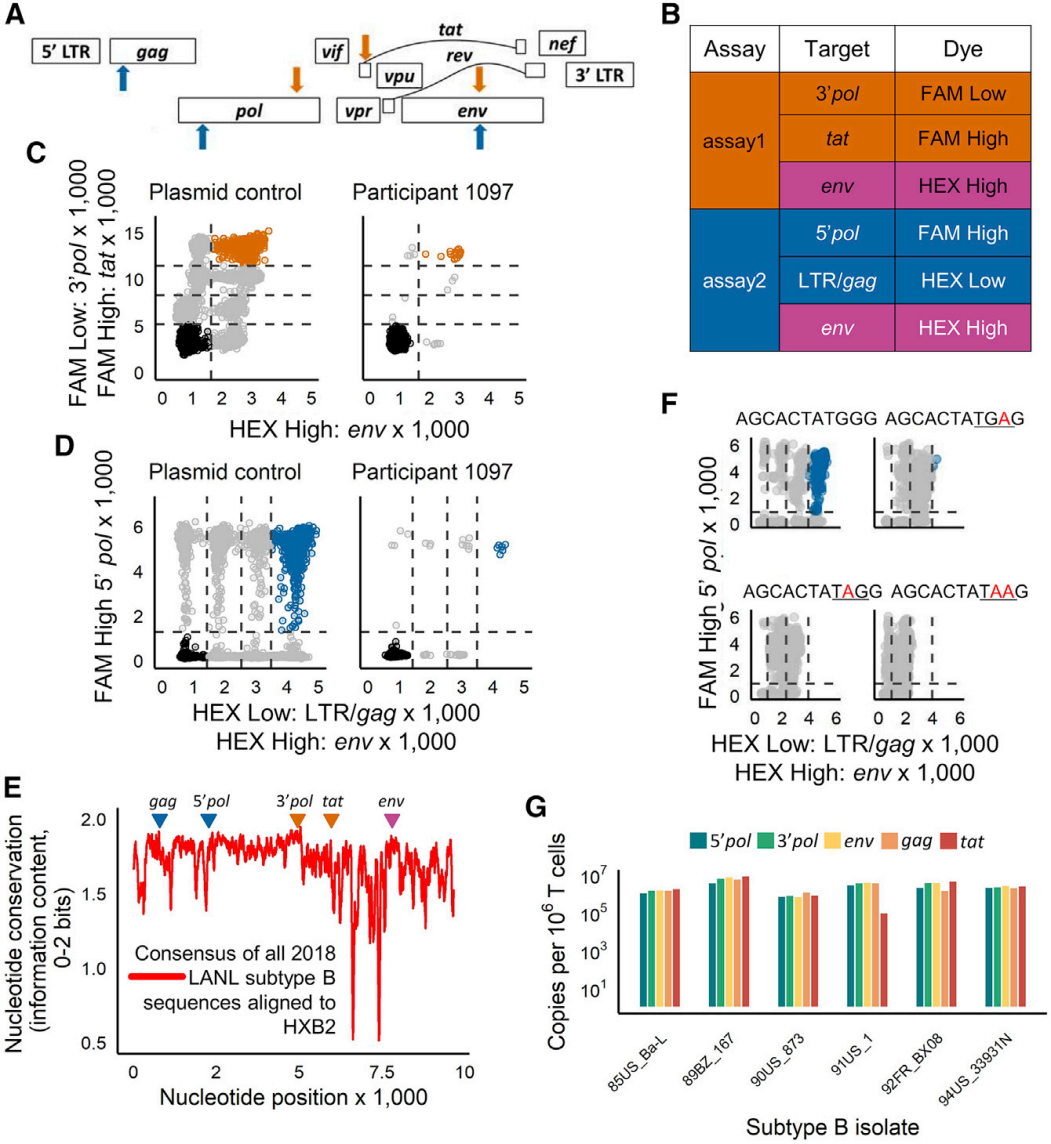
Design of the two HIV-1 triplex ddPCR assays (A) Primer/probe locations within the HIV-1 genome. Orange arrows, assay1 targets; blue arrows, assay2 targets. (B) Probe dyes and targets that constitute the two assays. Pink shading signifies the common env target. (C) ddPCR results from assay1 run on a plasmid control (plasmid mixtures of all possible single, dual, and triple combinations of all assay target regions) and participant 1097 (DNA extracted from CD4+ T cells isolated from an HIV-infected patient on ART). Orange dots indicate droplets positive for all three targets in assay1. (D) DNA templates as in (C). ddPCR results from assay2. Blue dots indicate droplets positive for all three targets in assay2. Three replicate wells were run for each HIV assay, and droplet counts were pooled. (E) Nucleotide conservation based on available subtype B HIV genome sequences from the Los Alamos National Laboratory (LANL) database aligned to HXB2. Triangles indicate position of the five probes. (F) Impact of hypermutation in the env probe sequences on ddPCR assay performance. A shift in HEX signal occurs when env plasmid sequences with introduced stop codons are used as the ddPCR template. Each test plasmid sequence, corresponding to coordinates 7352-7362 in HIV-1 NC_001802 (env), is indicated above its respective plot. Red bases show where G was substituted by A in the plasmid. These G-to-A hypermutations introduce stop codons (underlined). The upper left plot represents the pattern when the probe matches the plasmid coding strand sequence. The other three plots show the consequence of hypermutations on env target detection. The depicted plots are representative of 7 replicates of assay2. (G) DNA from PBMCs infected with five distinct HIV-1 subtype B viral isolates (indicated on the x axis) was quantified with both assay1 and assay2. Shown are the total number of copies detected for each target region. For each isolate, 2–4 replicate wells each were run for assay1 and assay2. One or 2 wells were run for the corresponding reference assay.

Our protocol for quantification of intact HIV-1 proviral copies consists of 2 triplex ddPCR assays, which together measure five targets in the HIV genome: one target (*env*) is repeated in both HIV assays (Figures 1A and 1B). The three HIV-1 targets in assay1 are in the 3′ end of *pol* (fluorescein amidite [FAM] low), in *tat* (FAM high), and in *env* (hexachlorofluorescein [HEX] high) and are spaced over approximately 3 kbp when aligned to HIV-1 NC_001802. The three HIV-1 targets in assay2 are in the long terminal repeat (LTR)/*gag* region (HEX low), the 5′ end of *pol* (FAM high), and in *env* (HEX high) and are spaced over approximately 7 kbp. Two of the three targets in each assay use the same dye for probe detection, but at different concentrations, to enable separation of different targets on an x/y plot of fluorescence amplitudes. This allows us to quantify droplets containing different combinations of targets (0, 1, 2, or 3 targets; Figures 1C and 1D).^19^ The *env* primers and probe are the same in assay1 and assay2, with nearly identical *env* performance between the two assays in 201 clinical samples (Figure S1A). Moreover, the failure rates of the five primer/probe pairs to detect a target, i.e., where a sample was entirely negative for a target, were extremely low in these clinical samples: *gag* 0.5%; 3′*pol* 1%; *env* 3.1% in both assays; *tat* 3.6%; and 5′*pol* 6.3% (Figure S1B).

The specific primers and probes were selected by analyzing sequences from the Los Alamos National Laboratory (LANL) database (https://www.hiv.lanl.gov) to include conserved and, for *env*, hypermutated regions. We also analyzed the published clade B sequences from the HIV Proviral Sequence Database (PSD)^20^ to target regions reported to contain deletions, to minimize mistaking incomplete viral genomes as intact. For example, the selected *env* region frequently contains deletions (74.6% of viruses in the database). Thus, importantly, when present and not hypermutated, the selected regions are relatively conserved across all available clade B sequences (Figure 1E).^21^, ^22^, ^23^, ^24^ To improve specificity for the HIV-1 gene targets, we incorporated locked nucleic acids (LNAs)^25^ into the probe sequences (Table S1).

The *env* probe detects hypermutated sequences as defective. The probe sequence for *env* detection in both HIV-1 triplex assays contains the sequence TGGG at its 3′ end (Table S1), with T and the first G being LNA. The complementary, intermediate, single-stranded *env* DNA sequence ACCC is a known target for cytidine deamination by APOBEC3G/3F, which causes G-to-A hypermutations in the coding proviral DNA strand. These mutations can lead to HIV-1 inactivation by introduction of stop codons (TAG, TGA, and TAA). To test whether our assay would correctly identify proviral sequences with stop codons at this site as defective, we tested three plasmids with such G-to-A mutations in the coding strand (TAGG, TGAG, and TAAG) and the original plasmid (TGGG; Figure 1F) with both assay1 (5 replicates) and assay2 (7 replicates). HEX fluorescence was strongly reduced for the three G-to-A mutated plasmids compared to the original plasmid, eliminating the triple-positive population. Thus, both HIV-1 triplex assays correctly identify proviral sequences with inactivating G-to-A hypermutations in the *env* probe-binding site as defective.

To assess the ability of our primer/probe sets to work across the expected diversity in subtype B sequences, we tested our assay on DNA from peripheral blood mononuclear cells (PBMCs) we infected with six subtype B viruses from the NIH International Panel of HIV-1 isolates.^26^ Because the DNA was from short-term virus cultures, we expected deletions in the sequences to be rare and quantification of all 5 target regions to be equivalent. Indeed, quantifications across the 5 target regions were within an average of 1.7-fold (geometric mean; range 1.3–41.8; Figure 1G). In one of the six subtype B sequences tested, 91US_1, the quantification of *tat* was an average of 37-fold lower than the 4 other target regions, suggesting that diversity in the *tat* primer/probe binding sites may result in an underestimation of intact proviral DNA for some samples when using assay1.

#### Quantification of T cells and correction for DNA shearing

**Figure 2:**
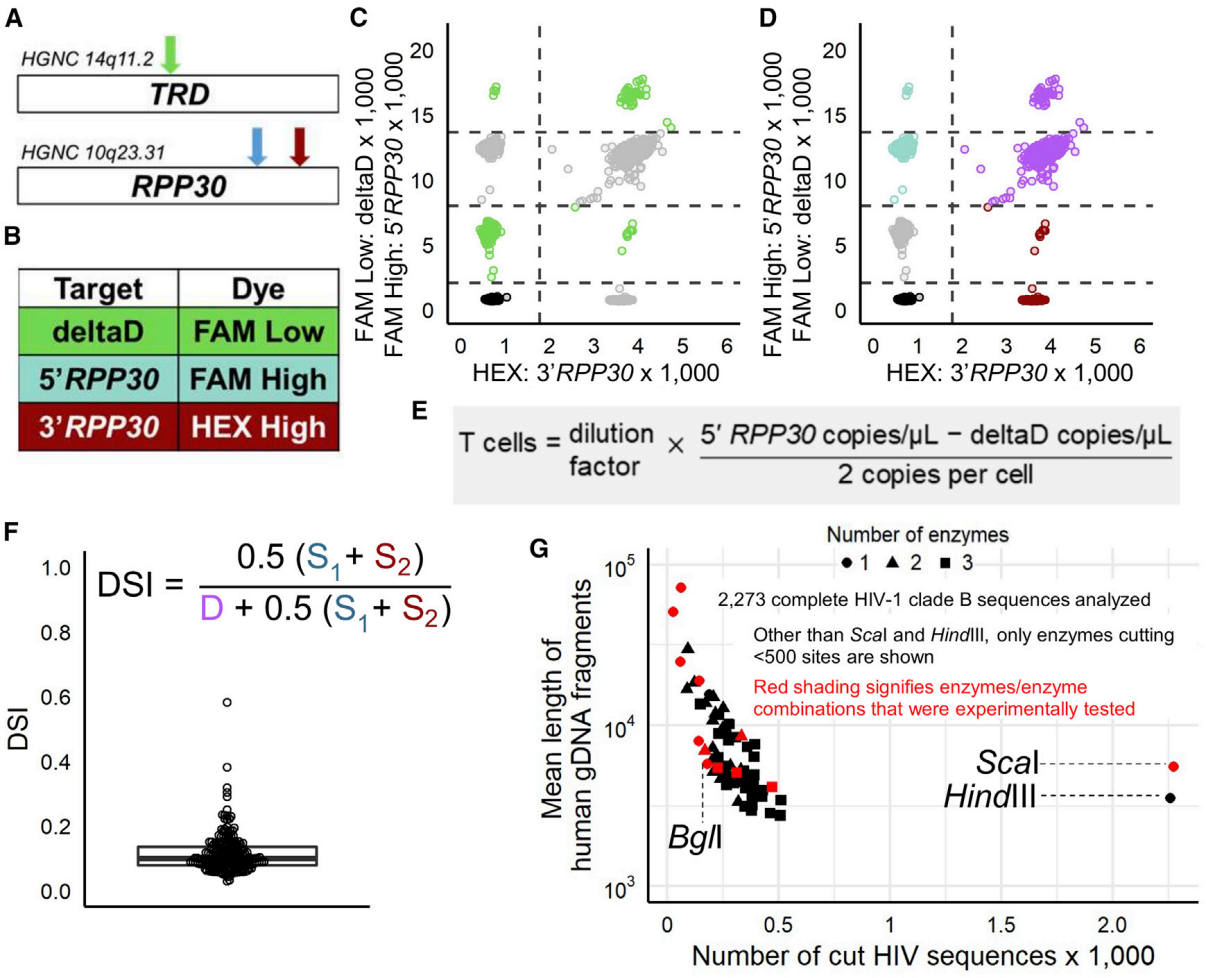
ddPCR reference assay (A) Target locations for the ddPCR reference assay within the human genome. The D region target in the T cell receptor gene (*TRD*) (green arrow) is located on chromosome 14 and is lost during T cell receptor rearrangement (“deltaD”). Thus, the assay directly quantitates all *non*-T cells. The two *RPP30* targets are located ~11 kbp apart from each other within the *RPP30* gene on chromosome 10 (blue and red arrows). (B) Probe targets and corresponding dyes. (C) Representative PBMC sample indicating droplets positive for the deltaD target (green dots), used to estimate non-T cell numbers. (D) Same plot as (C) but indicating droplets positive for only the 5′*RPP30* (blue dots), only the 3′*RPP30* target (red dots), or both *RPP30* targets (purple dots). The *RPP30* targets are analyzed independently from the deltaD target to quantify shearing and total cells. (E) Formula used to calculate the number of total T cells. Division by 2 is necessary because each cell contains two gene copies. Dilution factor signifies dilution of the test sample for cell counting relative to the template for the HIV-1 ddPCR assay, which is undiluted. (F) DSI distribution for 225 PBMC-derived CD4^+^ T cell samples. The formula for the DNA Shearing Index (DSI), the DSI-corrected number of triple-positive proviral copies, is given, where D represents the count of droplets positive for both *RPP30* targets (i.e., double positive) and S_1_ and S_2_ represent counts of droplets that are single positive for the 5′*RPP30* and 3′*RPP30* targets, respectively. (G) *In silico* analysis of the number of cutting sites in the HIV genome and mean human gDNA fragment length resulting from digestion by individual or combinations of commonly available restriction enzymes. The red symbols indicate those enzymes we subsequently tested *in vitro*. Two to four replicate wells were run for each test. *Sca*I and *Hind*III are typically recommended for digestion of gDNA prior to ddPCR. *Bgl*I yielded the best compromise between cutting very few HIV-1 sequences in the LANL HIV sequence database and cutting the human genome into ideal fragments for droplet generation, averaging ~6 kbp.

We designed a reference ddPCR assay that simultaneously allows normalization of HIV-1 proviral copy numbers to total T lymphocytes and a correction for DNA shearing (Figures 2A–2F). For samples where CD4^+^ T cells cannot be purified prior to DNA isolation, such as tissue specimens, quantification of the number of T cells by ddPCR is beneficial. Additionally, CD4^+^ T cell purification procedures, although very effective, do not yield completely pure populations, so quantification of T cells at the DNA level can improve accuracy. Thus, we used a previously published assay to probe a region in the *TRD* (T cell receptor D) gene that is excised during T cell receptor gene rearrangement in maturing T cells and is therefore only present in non-T cells (“deltaD”).^18^ We simultaneously targeted the *RPP30* gene, which is present in all cells, in a multiplexed ddPCR assay to quantify the total cell number in a sample, subtracted the non-T cell number, and then normalized provirus copies to 10^6^ T cells (Figure 2E).

DNA shearing during nucleic acid isolation can lead to undercounting of intact HIV genomes if HIV proviral DNA is fragmented. We measure shearing by incorporating two targets in the *RPP30* gene (5′*RPP30* and 3′*RPP30*) that are ∼11 kbp apart (slightly longer than the length of an intact HIV-1 proviral genome) into the ddPCR reference assay. The counts of droplets containing only one *RPP30* target provide a proxy for the fraction of template genomic DNA (gDNA) that was sheared into fragments of 11kb or less.^17^ A similar fraction of DNA shearing is expected for the ∼10-kbp HIV genome. Thus, we can use the number of *RPP30* single- and double-positive droplets in the reference assay to calculate a DNA shearing index (DSI), which is the probability that a template was sheared, using the formula in Figure 2F. The true number of “intact” templates is equal to the observed HIV assay output, divided by the fraction of templates that were *not* sheared (1-DSI). Therefore, corrected triple-positive copies = observed triple positive copies/1-DSI.

In addition to correcting for DNA shearing, we optimized our method for DNA isolation to minimize shearing in the first place. We compared several methods, including dialysis based (Megalong, G-Biosciences; RecoverEase, Agilent), magnetic bead based (MagAttract, QIAGEN), salting-out/alcohol precipitation (Gentra Puregene, QIAGEN; MasterPure, Lucigen), column based (QIAamp, QIAGEN), phenol-chloroform extraction,^27^ and a method described in the literature that uses guanidinium salts to lyse cells and separate cell proteins from nucleic acids, followed by precipitation with isopropanol and sodium acetate to isolate the genomic DNA.^28^ Selecting the latter method for our DNA isolation, the mean and median DNA shearing indices of our samples (0.10 and 0.11; range 0.03–0.59; Figure 2F) were markedly lower than those reported in a similar study using column extraction (∼0.30 and ∼0.30; range 0.22–0.53; Extended Data Figure 7 in Bruner et al.^17^).

#### Benefit of selective DNA digestion

Highly viscous gDNA, as isolated using our method, can lead to poor droplet formation during the ddPCR process; therefore, a restriction enzyme digestion targeting a commonly occurring recognition sequence is generally recommended to improve droplet formation. However, preserving intact HIV-1 sequences is essential to our assay. Thus, we performed an *in silico* analysis to determine which enzyme or combinations of up to three enzymes cut the human genome at regular intervals but would not cut within the integrated HIV-1 genome or within the reference gene *RPP30*.^21^^,^^29^, ^30^, ^31^, ^32^ Using the New England BioLabs database (https://www.neb.com/tools-and-resources/selection-charts/dam-dcm-and-cpg-methylation), we analyzed 168 restriction enzymes for their cut site sequences within 2,273 complete HIV-1 clade B sequences in the LANL HIV sequence database. We also eliminated enzymes with cut sites detected between the two primer/probe sites in *RPP30*, using human gDNA sequences from the University of Santa Cruz (assembly hg38, Dec. 2013 release, accessed via R package BSgenome.Hsapiens.UCSC.hg.38 or http://genome.ucsc.edu/).^33^ Based on our *in silico* analysis (Figure 2G), we selected the *Bgl*I enzyme, because it is predicted to cut between our outer target regions in only 7.9% of clade B viruses in the sequence database, while at the same time cutting the human genome into suitable fragments of an average length of 5,723 bp (range 2,749–71,633). The *Bgl*I recognition sequence is also absent in the ∼11-kbp region between the two target sites of the *RPP30* reference gene. Experimental comparisons to other enzymes and enzyme combinations demonstrated that *Bgl*I indeed left >92% of probed HIV-1 genomes intact (Figure 2G). Lastly, we formally demonstrated that low-shearing DNA isolation followed by controlled digestion using *Bgl*I is superior (median 54.7% *RPP30* double positive, n = 16) to high-shearing DNA isolation without enzyme digestion (median 14.5% *RPP30* double positive, n = 16; Figure S2).

#### Assay validation

**Figure 3:**
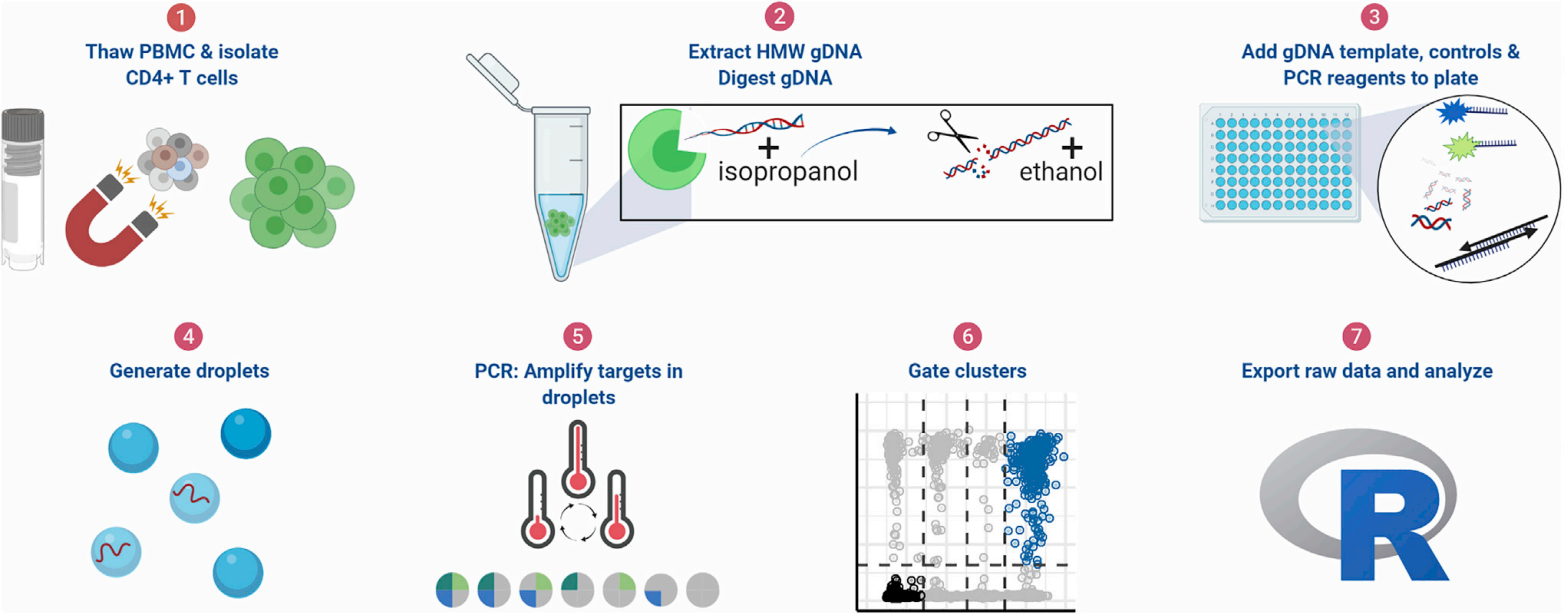
ddPCR protocol workflow (1) Thaw cryopreserved cells and isolate CD4^+^ T cells. For tissue samples, CD4^+^ T cells were not isolated. (2) Extract high molecular weight genomic DNA (gDNA) using guanidinium salts and isopropanol precipitation and then digest gDNA with the restriction enzyme *Bgl*I and precipitate the DNA with ethanol. (3) Add specimen gDNA, control plasmids, and PCR reagents to plate. (4) Generate droplets. (5) Conduct PCR reaction for amplification of targets in droplets. (6) Gate populations in QuantaSoft AP. (7) Analyze results in R.

The resulting intact proviral DNA ddPCR protocol includes thawing the patient sample, the option of separating the T cells, extracting and digesting the gDNA, adding the two triplex HIV ddPCR assays and the reference assay reagents and template to the plate, generating the droplets, amplifying the targets by PCR, and analyzing the data. This workflow is depicted in Figure 3 and applies to all subsequent experiments.

#### Specificity or limit of blank (LoB)

We tested the specificity of our assay using three types of negative control samples: (1) purchased HIV-negative PBMC gDNA from a mixture of donors (Promega G3041; 90 repeat tests); (2) gDNA isolated in our laboratory from 10 individual HIV-negative CD4^+^ T cell donors (29 repeat tests); and (3) gDNA isolated in our laboratory from the Jurkat T cell line (30 repeat tests).^34^ In a total of 149 negative control tests for each HIV-1 triplex assay, we detected only a single triple-positive droplet by assay1 and none by assay2. We conclude that both triplex assays are highly specific. Because both assays must be positive to conclude presence of intact provirus, we set the LoB for the protocol at zero triple-positive copies (Table S4).

#### Sensitivity, limit of detection (LoD), and precision

To determine the sensitivity and LoD of triple-positive copies, we prepared samples of HIV-negative Jurkat cells with spiked-in HIV^+^ J-Lat 8.4 T cell clones that contain a single full-length copy of HIV per cell, resulting in 0–5,000 HIV-positive cells per 10^6^ Jurkat cells.^35^ We performed three technical replicates of each of the two HIV-1 triplex assays and two replicates for the reference triplex assay (1:100 dilution of template for the reference assay to avoid saturation). Each HIV-1 PCR test contained DNA from an average of ∼245,000 cells (95% confidence interval [CI] 2.2 × 10^5^–2.7 × 10^5^). We repeated this experiment 14 times (Table S5). At ∼122.5, 24.5, 12.25, 1.225, and 0.245 HIV-positive cells per reaction, 14/14, 13/14, 12/13, 4/14, and 1/14 reactions detected triple-positive copies, respectively, demonstrating the high sensitivity of the protocol. We performed a probit analysis to calculate the LoD for which 95% of true triple-positive samples would be correctly identified as triple positive by our protocol. We determined the LoD to be 24 triple-positive copies per 10^6^ cells if 10^6^ cells are tested. If fewer cells are tested, the LoD increases; if more cells are tested, it decreases. For example, if only 250,000 cells are tested, the LoD is 96 (24 × 1,000,000/250,000). To gauge the precision of the two assays, we prepared a batch of 1,000 J-Lat 8.4 cells spiked into 1 × 10^6^ Jurkat cells (ratio 1:1,000) and ran aliquots on 22 separate occasions for both HIV-1 triplex assays. Mean triple-positive provirus-containing cells by assay1 were 935.8 (95% CI 759.8–1,112; coefficient of variation [CV] 42.4%) and by assay2 1,088 (95% CI 882.7–1,293; CV 42.5%), showing congruence between the two assays and satisfactory precision.

#### *In silico* evaluation of ddPCR protocol performance

**Figure 4:**
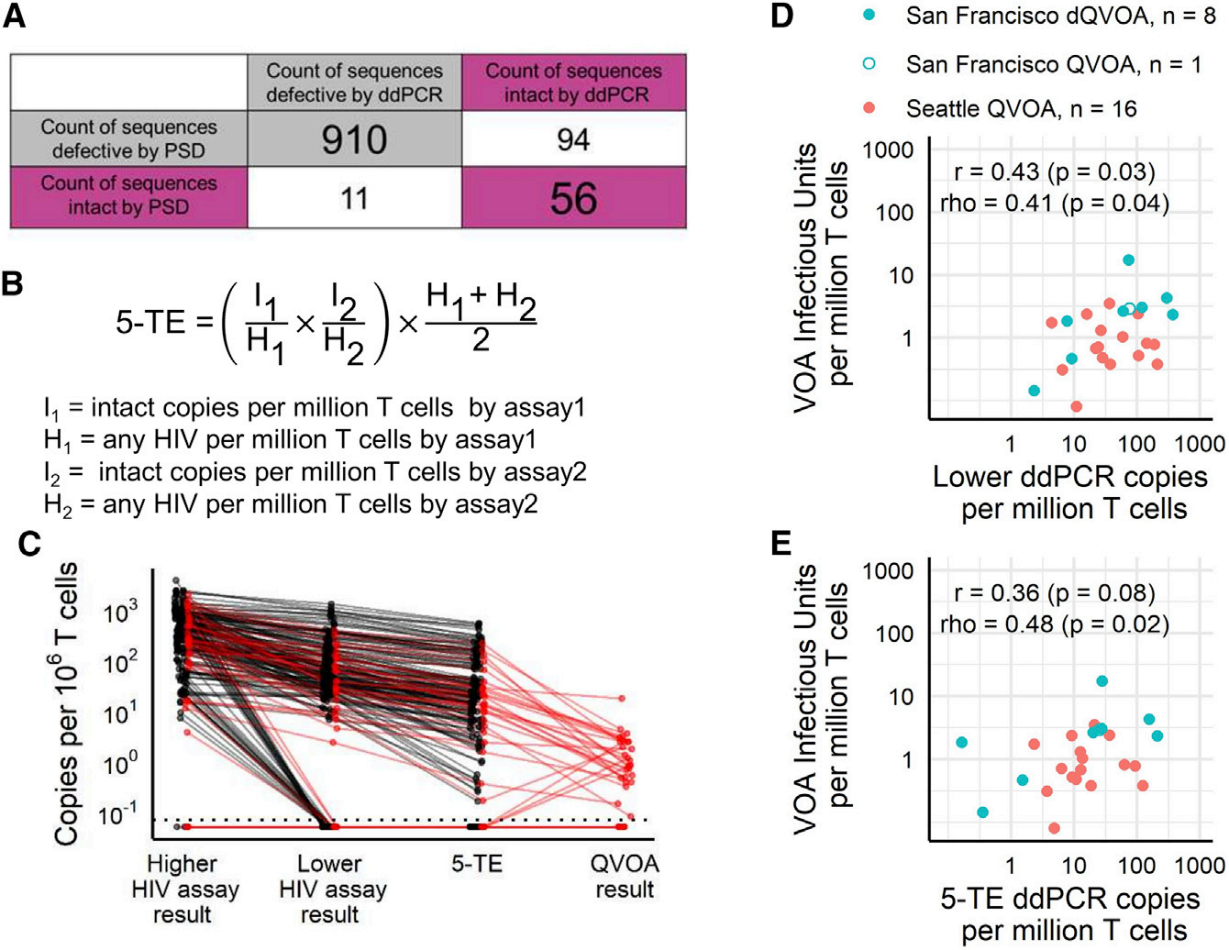
Assay validation and estimation of true intactness (A) 2 × 2 table showing the number of sequences with predicted intact or defective calls using our protocol and the calls based on sequence analysis in the Proviral Sequence Database. (B) Equation to calculate the expected number of HIV genome copies per 10^6^ T cells that would give triple-positive results from both HIV assays (5-target estimate [“5-TE”]): the probability of intactness by assay1 *and* intactness by assay2, multiplied by the average number of HIV copies (both intact and defective) that were detected across the two assays. (C) Approximation of true provirus intactness using the ddPCR protocol and relationship to quantitative viral outgrowth assay (QVOA). Shown are the lower and higher of the two HIV assay results (both n = 192), the 5-TE n = 192), and the QVOA result where available (red lines; n = 35). (D and E) Correlation between QVOA or dQVOA and the lower of the two assays (D) or 5-TE (E). Not included are samples where QVOA or ddPCR results were zero. Spearman’s rho and Pearson’s r are given, with p values resulting from tests of the null hypotheses that there is no monotonic (Spearman) or linear (Pearson) relationship between the two parameters. For the UW-CFAR_QVOA cohort (n = 14 PLH), two samples were tested at two different dilutions. For the San Francisco cohort (n = 9 PLH), one sample did not have a dQVOA result but did have a QVOA result (open blue circle). All tests were done with three replicate wells for the HIV assays and two replicate wells for the reference gene assay.

As an *in silico* test of the specificity and sensitivity of our ddPCR protocol, we “tested” our assays against published clade B sequences from the PSD.^20^ Based on our and others’ experience, we assumed that each primer/probe pair would recognize a published target region if the sequence had no more than five mismatches from our assays’ primer sequences and zero mismatches from the probes. We classified each PSD sequence as intact by ddPCR if all 5 primer/probe pairs would recognize it and as defective if this was not the case. We also classified all PSD sequences as intact and defective by sequencing, using criteria described by the Pro-Seq IT tool associated with the PSD database, which include intactness thresholds for sequence length, mutations, and deletions.^13^^,^^36^^,^^37^ We then compared the two classifications (ddPCR and sequencing) to quantify agreement. Of the 1,071 PSD sequences, 966 sequences (90.2%) agreed between the PSD algorithm and our ddPCR protocol (Figure 4A). Eleven sequences (∼1%) were considered intact by the PSD algorithm but defective by our assays. These differences were all due to >5 mismatches in primer sequence, with 10 of these 11 in *tat* primer sequences, and 8 of these 10 due to mismatches in the same location. Ninety-four sequences (∼8%) were considered defective by the PSD algorithm but intact by our assays. The PSD algorithm calls 72 of these 94 defective due to a missing or mutated major splice donor site at HXB2 724–745. In 71/94 there also was a >7-nt deletion in the packaging signal. Both regions are not covered by our five primer/probe sets. Other differences were due to premature stop codons in *gag* or *pol* or long deletions in *gag*, *pol*, and *env.* Overall, the *in silico* evaluation demonstrates that our ddPCR protocol approaches the fidelity of complete proviral sequencing, misclassifying fewer than 10% of sequences.

#### Combining both HIV-1 ddPCR assays for a more-accurate estimate of proviral intactness

To conclude that a specimen contains intact provirus, both HIV-1 triplex assays must be positive. If this is the case, multiplying the intactness probabilities (i.e., proportions of intact/total HIV-1 copies) of the two assays gives the probability that detected proviruses in a specimen contain all five primer/probe regions (see formula in Figure 4B). We call this estimate of very likely intact proviruses the 5-target estimate or “5-TE,” and the respective assay the 5-target intact proviral DNA assay (5T-IPDA). Across 151 patient samples from three cohorts, 5-TE copy numbers ranged from 0.2 to 526.2 per million T cells (mean 64.8; median 19.7;Figure 4C), compared to a range of 2.3–1,280 (mean 157.8; median 60.0) copies reported by the corresponding lower HIV-1 triplex assay (i.e., either assay1 or 2, depending which gave the lower result) and 10.9–2,373 (mean 537.1; median 385.0) copies reported by the higher HIV-1 triplex assay. On average (geometric mean), the 5-TE reported 3.6-fold fewer copies than the lower HIV-1 triplex assay and 16.3-fold fewer copies than the higher HIV-1 triplex assay.

Next, we wanted to assess how the proportion of intact proviruses reported by our protocol’s 5-TE results compares to the gold standard of proviral sequencing studies, which had estimated that around 2%–5% of proviral DNA sequences are intact and reflect chromosomal integration of replication-competent virus.^4^^,^^12^^,^^14^^,^^17^ Across all blood samples we tested (n = 192), our protocol reported a mean percentage of intact proviruses of 3.2% (range 0–41.87; 95% CI 2.4–4.0).

#### Comparison with quantitative viral outgrowth assay

To compare our protocol to standard approaches quantifying the replication-competent reservoir, we used our assays to quantify provirus in samples from two participant cohorts of people living with HIV (PLH) that had previously been measured by QVOA in other cohort studies: one cohort from Seattle (n = 16 from 12 donors) and the other from San Francisco (n = 9 from 6 donors). To achieve adequate sample size to assess correlation between ddPCR and QVOA, we combined the cohorts. Our assay results were significantly correlated with the QVOA results, regardless of whether we used the lower of the two triplex ddPCR assay results or the 5-TE results (Figures 4D and 4E). We observed a roughly log-log linear relationship between the lower result versus QVOA (rho = 0.41; p = 0.04) and between the 5-TE versus QVOA (rho = 0.48; p = 0.02). The 5-TE reported on average 12.1 times higher copies per million CD4^+^ T cells (range 0.09–324.9) than the QVOA results (Figure 4C). The only other HIV-1 multiplexed ddPCR assay published reported intact provirus copy numbers to be on average approximately ∼78.8-fold higher than QVOA (range 1.5–2,941.4).^17^

Of note, 8/25 QVOAs were done using a protocol employing *ex vivo* differentiation of resting CD4^+^ T cells into effector memory cells prior to T cell activation (differentiation QVOA [dQVOA]).^3^ QVOA and dQVOA results were highly correlated in prior comparisons (Figure 3b in Wonderlich et al.^3^; Spearman's rho = 0.85; p = 0.00004). For the eight dQVOAs, the 5-target ddPCR estimate was only 4.79-fold higher (mean; range 0.09–90.94).

### Use of the multiplex ddPCR assay to quantify the HIV-1 reservoir in patient samples

#### Intact provirus quantification in longitudinal blood samples from people living with HIV on ART

**Figure 5:**
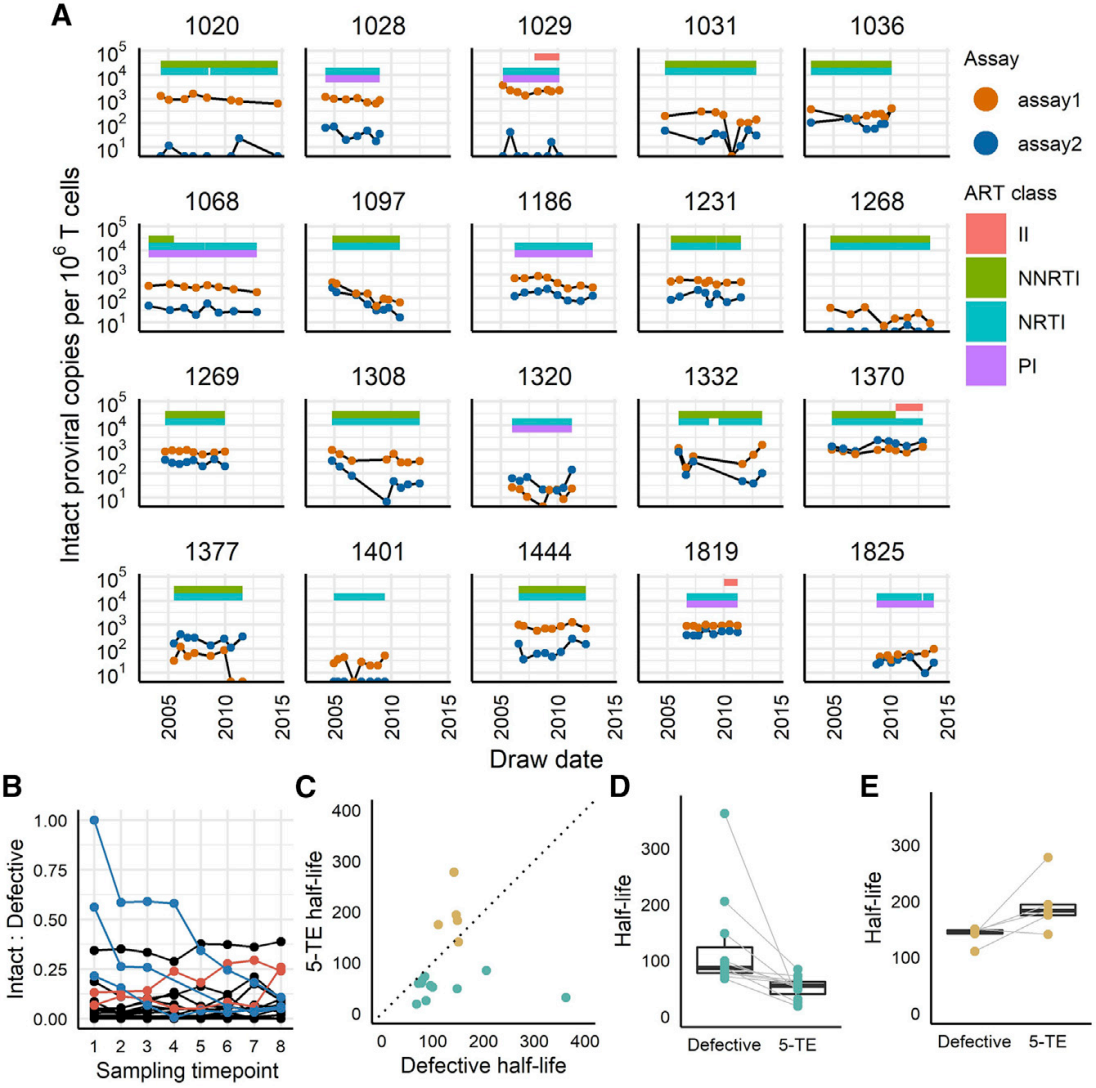
Longitudinal analysis of 20 PLH (A) Longitudinal testing of 20 PLH in Seattle. CD4^+^ T cells negatively selected from cryopreserved PBMC samples from 20 participants in the UW-CFAR_KINETICS cohort were tested at 8 time points (n = 17), 7 time points (n = 1), or 6 time points (n = 2) over a period of 4.5 – 10 years on ART. Shown are the results for intact proviral copy numbers measured by HIV-1 multiplex assay1 (orange circles) and assay2 (blue circles). All tests were done with three replicate wells for the HIV assays and two replicate wells for the reference gene assay. Data points falling on the x axis represent “undetectable.” Colored bars represent the participants’ drug regimens, categorized by class of drug action, over time. II, integrase inhibitors; NNRTI, non-nucleoside reverse transcriptase inhibitors; NRTI, nucleoside/nucleotide transcriptase inhibitors; PI, protease inhibitors. (B) Ratio of intact (5-TE) to defective proviral copies at each sampling time point for all 20 participants. Blue lines indicate a downward trend, and red lines indicate an upward trend. (C) Half-lives (months) of defective versus intact (5-TE) proviral copies (n = 16; 4 participants had no decay in intact copies). Teal circles indicate participants with intact (5-TE) half-life <10 years; light brown circles indicate >10 years. The diagonal dotted line signifies equal 5-TE and defective half-lives. (D) Defective and intact (5-TE) provirus half-lives for participants with 5-TE half-life <10 years. Boxes and whiskers: median; interquartile range, <1.5 × IQR and >1.5 × IQR. (E) Defective and intact (5-TE) provirus half-lives for participants with 5-TE half-life >10 years.

We quantified intact proviruses in 6–8 longitudinal samples from 20 ART-suppressed participants across a range of 4.5–10 years on ART (n = 157 blood draws; Figures 5 and S3–S5; Table S6). Across all samples from all participants, reservoir size was an average of 538.8 (assay1; 95% CI 449.5–628.1), 186.4 (assay2; 95% CI 126.7–246.2), and 56.5 (5-TE; 95% CI 40.7–72.4) provirus copies/10^6^ T cells (Figure 5A; 5-TE not separately shown). Assay2 reported a higher value than assay1 in only 22 samples (14%), which came from three participants. In two of these (IDs 1370 and 1377), assay2 was higher at all time points, and in the third participant (1320), assay2 was higher at 6/8 time points and minimally lower at the remaining two time points (1.2 and 0.4 copies/10^6^ T cells lower than assay1). Thus, the triplex assay that reported a higher number of intact (triple-positive) copies/10^6^ T cells remained largely consistent over time within each participant. Zero counts of intact provirus were reported by at least one assay in 31/157 (19.7%) samples and by both assays in 2/157 (1.3%).

The ratios of intact (by the 5-TE) to defective proviruses were relatively stable over time in 15/20 individuals (Figure 5B). However, 5/20 PLH showed a trend over time, with 3 strongly trending downward from high initial intactness and 2 slightly trending upward. For one participant (ID 1370), the intact/defective ratio remained above 0.25 for all 8 samples during the sampling period, despite 1370’s 27 plasma viral load measurements during the sampling period all being undetectable. Lastly, we determined the 5-TE reservoir half-lives for these 20 PLH. Four PLH did not show any decay. In the other 16 PLH, the mean intact reservoir half-life was 96.1 months (∼8 years; range: 18–278 months; Figure 5C). In contrast, the mean half-life of the defective reservoir was 250 months (∼21 years; 68–2,166 months; 5-TE versus defective slope; p = 0.09 by Mann-Whitney test). Eleven of these 16 PLH had 5-TE half-lives <10 years; in all 11 PLH, 5-TE half-lives were shorter (mean 51.7 months; range 18.2–84.5 months) than defective half-lives (mean 124.9 months; range 67.7–361.6 months; Figure 5D). For the 5 PLH with 5-TE half-lives >10 years, defective half-lives were longer (Figure 5E).

#### Intact provirus quantification in mucosal samples

**Figure 6:**
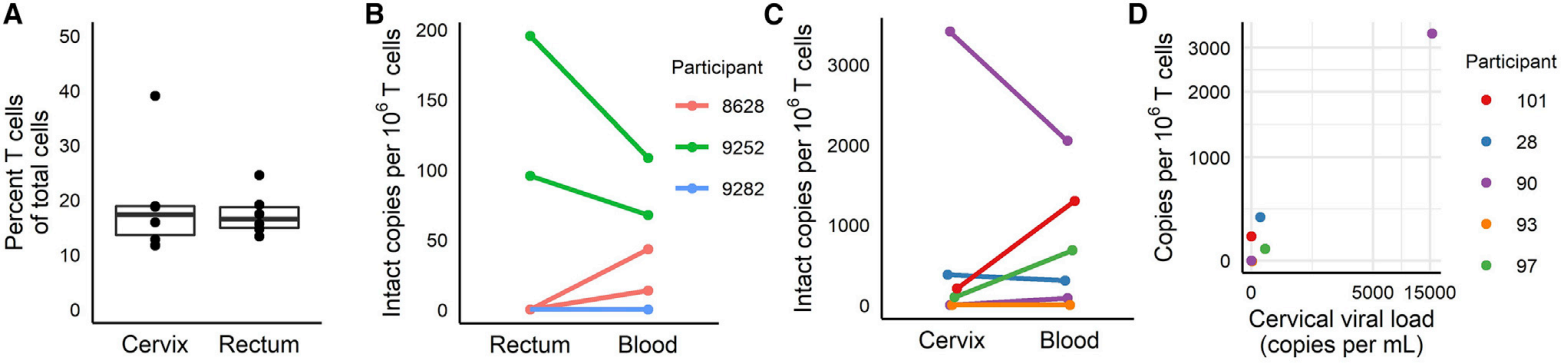
Paired testing of mucosal and blood specimens from 8 PLH (A) Percent T cells of total cells in rectal and ectocervical biopsies; n = 6 per tissue type. Boxes and whiskers: median; interquartile range, <1.5 × IQR and >1.5 × IQR. (B) DSI-corrected intact proviral copies per 1 million T cells in PBMCs and rectal biopsies. (C) DSI-corrected intact proviral copies per 1 million T cells in PBMCs and cervical biopsies. (D) Correlation between viral loads measured from cervicovaginal lavage versus intact proviral copies in cervical T cells by ddPCR. Axes in (D) are pseudo-log10 scaled, transitioning to a linear scale approaching zero. Samples from the Discordant Shedding Cohort were run with 6 replicate wells for assay2 (assay1 was not performed on these samples) and two replicate wells for the reference assay. For the ACTU-2100 Cohort, we ran 3–11 replicate wells for assay2 and 2–6 wells for the reference assay, depending on how much gDNA was available.

To compare intact proviral copy numbers between circulating and mucosal T cells, we tested rectal biopsies and PBMCs in 3 men living with HIV (ACTU-2100 Cohort) and cervical biopsies and PBMC in five women living with HIV (Discordant Shedding Cohort).^38^ For each of the three men, we extracted gDNA from paired blood T cells and rectal biopsies collected at 2 time points obtained 1 year apart. We saw more DNA shearing in the rectal biopsies (mean 0.4; 95% CI 0.3–0.6; SD 0.13) than in the blood T cells (mean 0.08; 95% CI 0.05–0.1; SD 0.12; paired t test; p = 0.001). On average, 17.4% (95% CI 13.2–21.7; SD 4.0) of all rectal cells were T cells (Figure 6A). Copies per million T cells of DSI-corrected likely intact provirus were detectable in both sample pairs from one participant and undetectable from the second participant (Figure 6B). In the third participant, we detected intact provirus in the blood, but not the rectal samples. For five women, we extracted gDNA from paired PBMC pellets and OCT-embedded ectocervical biopsies collected at the same clinic visit, with one of the participants providing 2 sets of paired samples from different time points (n = 6 sets of paired samples total). Again, the DSI was higher for ectocervical biopsies (mean 0.35; SD 0.22; 95% CI 0.12–0.58) than for PBMCs (mean 0.09; SD 0.04; 95% CI 0.05–0.13; p = 0.03; paired t test). A mean 19.5% (95% CI 9.0–30; SD 10.0) of all ectocervical cells were T cells (Figure 6A). After correction for DSI, copies of likely intact provirus averaged 683 per million T cells in the ectocervix (range 0–3,414; SD 1,345) and 738 per million T cells in PBMCs (range 0–2,052; SD 800; Figure 6C). The order from highest to lowest values was the same for ectocervix and PBMCs, except for one set of paired samples. We also measured viral RNA in cervicovaginal lavages (CVLs). Cervicovaginal viral loads and intact proviral DNA copies in cervical T cells were not statistically correlated (Spearman’s rho = 0.7; p = 0.12), but 5/6 were concordant when categorized into positive and negative specimens: 3/6 were positive for intact provirus in cervical T cells and viral RNA in CVL and 2/6 were negative for both. 1/6 was discordant: positive for intact provirus but negative for viral RNA (Figure 6D). Finally, we compared intact proviral copy numbers per million T cells between the paired mucosal and blood specimens from both mucosal cohorts combined (n = 12 pairs) and found no significant difference (p = 0.89; paired t test).

## Discussion

Clinical trials evaluating HIV cure strategies require accurate measurement of the size of the replication-competent HIV reservoir. The relevant unit is the number of HIV proviruses (defined as HIV genomes integrated into host-cell chromosomal DNA) that can produce infectious virions able to infect and complete replication cycles in new cells. HIV rebound following cessation of ART depends entirely on such replication-competent proviruses. Precise quantification of the replication-competent reservoir is difficult because *ex vivo* assays with patient cells cannot fully model the many functional components of a replication-competent virus *in vivo* (reviewed in Abdel-Mohsen et al.^39^). However, our 5T-IPDA, a highly multiplexed ddPCR protocol, which counts the number of proviruses containing 5 target regions, allows a closer approximation of the true quantity of replication-competent proviruses than either quantitative VOAs or other IPDA assays.^17^ The 5T-IPDA reports numbers that fall, on average, approximately 1-log above the results of quantitative VOAs performed on the same samples and nearly 1-log below the IPDA/QVOA ratio for a 2-target IPDA reported last year.^17^ This is encouraging, as there is broad consensus that quantitative VOA underestimates the number of replication-competent proviruses and the true number must therefore be higher,^4^^,^^40^ which was indeed the case for all 35 samples we tested by both assays. Simultaneously probing five targets in the proviral genome allows us to screen more of the genome for deletions. Thus, the 5T-IPDA minimizes the degree of overestimation of intactness that can occur if just one or two regions are examined, as reflected by our assay estimating intact provirus an average of 12.1 times higher than quantitative VOAs, compared to 78.8 times higher for the 2-target IPDA.^17^

The specificity of our 5T-IPDA is exceptional (0 false-positive intact proviruses in 149 negative controls). Sensitivity is also high, as the protocol reliably detected intact proviruses when ≤10 per reaction. By probing several proviral regions, our protocol is more sensitive than traditional single-target HIV DNA assays when measuring the total HIV-1 DNA (both defective and replication-competent proviruses). Figures 1C and 1D show that a single-target qPCR assay that, e.g., probes a conserved LTR/gag region, would miss a substantial proportion of defective proviruses. This enhanced sensitivity to detect HIV proviruses can be helpful when only a limited number of cells are available, for example, from infants. In patients off ART following a curative intervention, it could also be desirable to detect any HIV DNA as sensitively as possible, even after intact proviruses have fallen below detection.

Several additional findings support the quality and validity of our 5T-IPDA protocol. (1) All five primer/probe pairs quantify their respective HIV-1 clade B target sequences at similar levels (Figure 1G). (2) The overall failure rates of each of the five primer/probe pairs to detect their target sequences at all was low across all tested clinical samples (between 0.5% and 6.3%; Figure S1B). (3) The *env* probe performs identically between the two triplex assays (Figure S1A) and (4) correctly identifies G-to-A hypermutations introducing stop codons as defective (Figure 1F). (5) Gentle DNA isolation followed by selective DNA digestion results in substantially lower DNA shearing (mean DSI 0.10) compared to the DNA preparation method used for a previously reported IPDA protocol (mean DSI ∼0.30; Figure 2F), and minimizing DNA shearing likely improves the accuracy of quantifying intact proviruses.

An *in silico* analysis of our 5 primer/probe sequences found that they correctly identified >90% of 1,071 full-length clade B sequences from the PSD as intact or defective (Figure 4A). In addition, our 5T-IPDA results from 151 HIV^+^ PBMC samples from clade B virus-infected participants classified ∼4% of proviruses as intact, which is similar to the proportion of intact proviruses in prior reports that used proviral sequencing.^4^^,^^12^^,^^14^^,^^17^ Further, we found a significant correlation between our 5T-IPDA and QVOA measurements (Figures 4D and 4E).

In longitudinal data from 20 PLH, our protocol reported a faster decline of intact than defective proviruses, which agrees with recent reports using the 2-target IPDA.^41^^,^^42^ Similarly, we observed substantial variability in reservoir decay between individuals, with those having half-lives >10 years showing no acceleration of intact over defective decay (Figures 5B–5E). Our decay rates of intact proviruses were close to those reported earlier by QVOA,^43^, ^44^, ^45^, ^46^ on average 96.1 months for the 16/20 PLH with negative decay rates and 51.7 months for the 11 of these 16 with half-lives <10 years. Lastly, as previously reported,^6^ in 3 individuals with unusually high initial percentages of intact proviruses (>10%), defective proviruses accumulated rapidly relative to the intact ones.

There are few publications about the presence of replication-competent proviruses in tissues^47^, ^48^, ^49^, ^50^ and none comparing intact to defective proviruses in the mucosa. Thus, it remains unknown whether mucosal sites,^51^ especially the gut,^52^, ^53^, ^54^, ^55^ are clinically relevant reservoirs that cause intermittent plasma viremia during suppressive ART and/or viral rebound after ART cessation.^56^^,^^57^ Therefore, optimizing our protocol for use with tissue specimens was a crucial step to expand future reservoir studies beyond peripheral blood. One important component of this optimization is the ability to normalize proviral copy counts to the number of HIV target cells. Cellular composition can vary markedly between individuals, anatomical sites, and even multiple biopsies from the same tissue.^58^ With blood, we can purify CD4^+^ T cells before ddPCR. For tissues, this strategy requires dissociating the tissue and bringing the released cells into single-cell suspension prior to purification.^59^^,^^60^ Tissue dissociation protocols are laborious, pose biohazards, and suffer from procedural biases (for example, macrophages die more easily during cell isolation than T cells) and cell loss. Our reference ddPCR assay measures total T cell number (as well as DNA shearing) directly from the DNA, avoiding the need for cell purification.

Thus, our protocol will improve the ability to monitor the mucosal HIV reservoir. This could be especially important in cases where a curative regimen drives intact provirus counts in peripheral blood cells below the limit of detection, and it is desirable to survey accessible tissue sites, such as the rectal mucosa, for residual provirus. In our small study of mucosal specimens, we did not observe higher intact provirus frequencies in mucosal compared to peripheral blood T cells. However, much larger sample sizes will be necessary to compare proviral decay rates in peripheral blood and tissues and to determine whether disappearance of intact provirus from blood correlates with eradication from tissues.

### Limitations of study

Practically, our 5T-IPDA is only marginally more complicated to conduct than the IPDA with 2 targets. In addition to one HIV and one reference ddPCR reaction, it requires a second HIV ddPCR reaction, for a total of three parallel assays. However, digital PCR instrument technology is rapidly evolving and will soon include 4–6 separate fluorescence channels (compared to the 2-fluorescence channel detector we used for this study). This will enable multiplexing of our 5 primer/probe pairs into a single digital PCR reaction. In theory, even higher multiplexing will be possible, but careful mathematical evaluation should assess at which point the gain in precision to define a provirus as intact becomes so incremental that the added cost and complexity is not justified. In fact, the finding that our assay results fall squarely in between quantitative VOA and the 2-target IPDA indicates that a 5-probe assay may already be close to that threshold.

Although the ability to normalize the number of intact proviruses to the number of T cells without separating out the CD4^+^ T cells first is a distinct advantage of our protocol, we are only able to quantify total T cells and not specifically the CD4^+^ T cells. On the other hand, isolation of CD4^+^ T cells prior to DNA isolation misses HIV proviruses present in other cell types. A ddPCR protocol captures provirus in any cell type when DNA is isolated from bulk cells or tissues. This could be relevant for mucosal or central nervous tissues, where HIV-infected macrophages can be long lived and thus constitute part of the latent HIV reservoir.^61^

An important caveat to any IPDA is the potential for both over- and underreporting the true number of intact proviruses. Overreporting would occur when the integrated HIV genomes contain defects in regions not captured by the five primer/probe pairs, whereas underreporting would occur when any primer/probe pair fails to recognize a functional genomic region because of viral polymorphism. Both possibilities have been highlighted by recent reports. Gaebler et al.^62^ addressed the first possibility, and Kinloch et al.^63^ provided a comprehensive and thoughtful assessment how HIV sequence polymorphisms, especially also within an infected individual, may cause reservoir underestimation. Although the two errors could cancel each other out in population-wide analyses, in any given individual, these limitations may reduce the accuracy of reservoir estimation. Furthermore, an IPDA cannot be used to infer clonality or sensitively detect immune escape. Thus, at least for the time being, proviral sequencing should accompany important endpoints in HIV cure trials.

Of note, however, not even full-length sequencing of proviral clones can determine with certainty that a given provirus verified as genetically intact can produce infectious progeny. For example, successful transcription of a proviral genome depends on its specific integration site and orientation in the cellular genome.^64^, ^65^, ^66^, ^67^, ^68^ Our current ddPCR protocol does not assess proviral integration. However, novel digital PCR technology where DNA can be recovered following amplification and detection could enable proviral quantification and integration-site analysis from the same specimens. As a final limitation to our protocol, we developed it for counting intact and defective proviruses belonging to the subtype B strains predominant in the Americas and Western Europe,^69^ and it thus requires adaptions to work for other global HIV subtypes.

## STAR★Methods

### Key resources table

**Table:** 

REAGENT or RESOURCE	SOURCE	IDENTIFIER
**Bacterial and virus strains**		

90US_873	NIH HIV Reagent Program	Cat# ARP-11251
91US_1	NIH HIV Reagent Program	Cat# ARP-7686
92FR_BX08	NIH HIV Reagent Program	Cat# ARP-11420
94US_33931N	NIH HIV Reagent Program	Cat# ARP-11250
85US_Ba-L	NIH HIV Reagent Program	Cat# ARP-510
89BZ_167	NIH HIV Reagent Program	Cat# ARP-7692

**Biological samples**		

Peripheral blood mononuclear cells	Bloodworks Northwest	https://www.bloodworksnw.org

**Chemicals, peptides, and recombinant proteins**		

Buffer ATL	QIAGEN	Cat#19076
Buffer EB	QIAGEN	Cat#19086
Guanidine HCl	Research Products International Corp.	Cat#G49000-100.0CAS No. 50-01-1
3M Sodium Acetate	Invitrogen	Cat#AM9740CAS No. 127-09-3
Guanidine Thiocyanate	Chem Cruz: Santa Cruz Biotechnology Inc.	Cat#SC-202638CAS No. 593-84-0
KpnI-HF	New England BioLabs	Cat#R3142S
NcoI-HF	New England BioLabs	Cat#R3193S
NsiI-HF	New England BioLabs	Cat#R3127S
PacI	New England BioLabs	Cat#R0547S
SalI-HF	New England BioLabs	Cat#R3138S
SbfI-HF	New England BioLabs	Cat#R3642S
XmaI	New England BioLabs	Cat#R0180S
BssSαI	New England BioLabs	Cat#R0680
XhoI	New England BioLabs	Cat#R0146S
PspXI	New England BioLabs	Cat#R0656S
PmeI	New England BioLabs	Cat#R0560S
HindIII	New England BioLabs	Cat#R0104S
ScaI	New England BioLabs	Cat#R3122S
BglI	New England BioLabs	Cat#R0143S
Proteinase K, 20mg/mL, > 318mAu/mL at 30°C	QIAGEN	Cat#19131
AatII	New England BioLabs	Cat#R0117S
Pen Strep	GIBCO	Cat#15140122
L-glutamine	GIBCO	Cat#25030164
RPMI 1640 Medium, HEPES	GIBCO	Cat#22400089

**Critical commercial assays**		

ddPCR supermix for probes (no dUTP)	Bio-Rad	Cat#1863024

**Experimental models: cell lines**		

J-Lat 8.4 cell line	NIH HIV Reagent Program	Cat#9847-427; RRID: CVCL_8284
Jurkat E6-1 cell line	ATCC	Cat#TIB-152; RRID: CVCL_0367

**Oligonucleotides**		

HIV-1 primers/probes	Integrated DNA Technologies	Table S1
*TRD* gene primers/probes	Integrated DNA Technologies	Table S2
*RPP30* gene primers/probes	Integrated DNA Technologies	Table S3

**Recombinant DNA**		

pUCIDT_Amp	Integrated DNA Technologies	N/A

**Software and algorithms**		

Microsoft Office	https://www.microsoft.com/en-us/microsoft-365	N/A
Bioconductor	http://www.bioconductor.org	RRID: SCR_006442
Biorender	https://biorender.com/	N/A
R software 4.0	http://www.r-project.org/	RRID: SCR_001905
QuantaSoft AP 1.0.596	Bio-Rad	https://www.bio-rad.com/en-us/product/qx200-droplet-digital-pcr-system

**Other**		

PCR Plate Heat Seal, foil, pierceable	Bio-Rad	Cat#1814040
Bio-Rad PX1 PCR Plate Sealer	Bio-Rad	Cat#1814000
Wide bore p1000 pipette tips	Rainin	Cat#30389195
Wide bore p200 pipette tips	Rainin	Cat#30389217v

### Resource availability

#### Lead contact

Requests for resources and information should be directed to the Lead Contact, Florian Hladik (florian@uw.edu)

#### Materials availability

The plasmid sequences used for gating controls are available from AddGene with the following identifiers: 167347, Seattle_IPDA_control_1_001; 167348, Seattle_IPDA_control_6_002; 167349, Seattle_IPDA_control_9_003; 167350, Seattle_IPDA_control_14_004; 167351, Seattle_IPDA_control_17_005; 167352, Seattle_IPDA_control_29_006; 167353, Seattle_IPDA_control_32_007.

#### Data and code availability

The datasets generated from this study are available from the Lead Contact upon request, except the private health information of study participants.

### Experimental model and subject details

#### Human participant cohorts

See Table S6 for demographic details of human subject participants. In some cases, details were unavailable because they were not collected during the initial study. We used anonymized samples from five cohorts: UW-CFAR_KINETICS (Seattle, WA, USA; longitudinal peripheral blood mononuclear cell [PBMC] samples), UW-CFAR_QVOA (Seattle, WA, USA; comparison to QVOA), RAVEN (San Francisco, CA, USA; comparison to QVOA), ACTU-2100 (Seattle, WA, USA; PBMC and rectal samples) and the Discordant Shedding Cohort (Lima, Peru; PBMC and cervical samples) (Table S6). The UW-CFAR_KINETICS and UW-CFAR_QVOA samples were 34 PBMC samples from people living with HIV (PLH) on suppressive ART from a clinical cohort maintained by the University of Washington Center for AIDS Research. In 20 of these (UW-CFAR_KINETICS), we isolated CD4^+^ T cells from 6-8 longitudinal PBMC draws per donor donated over a period of 4.5-10 years. In the other 14 (UW-CFAR_QVOA), we used PBMC from a single time point per donor to measure provirus by QVOA and ddPCR. The RAVEN (Reservoir Assay Validation and Evaluation Network) samples included 9 PBMC samples from the RAVEN cohort of PLH. We isolated CD4^+^ T cells from the RAVEN PBMC to measure provirus by traditional QVOA, differentiation QVOA (dQVOA)^3^ and ddPCR. The ACTU-2100 cohort consisted of PLH on suppressive ART who participated in the AIDS Clinical Trials Unit 2100 study to use mycophenolate mofetil to reduce the HIV reservoir (NCT03262441). We used genomic DNA from CD4^+^ T cells and rectal biopsies for ddPCR. The samples were collected from three participants at two time points each. The Discordant Shedding Cohort group consisted of 5 PLH on ART from a study of residual genital shedding during antiretroviral therapy in Lima, Peru^38^. For 4/5 participants, we used paired cervical and PBMC samples from a single time point and samples from two time points (28 weeks apart) for the remaining participant.

#### Protection of human subjects

UW-CFAR_KINETICS and UW-CFAR_QVOA samples were obtained from the HIV Specimen Repository maintained by the Seattle Center for AIDS Research (CFAR). This repository is a collection of frozen plasma and PBMC specimens donated by HIV infected patients cared for at the University of Washington HIV outpatient clinics. Coded clinical data extracted from the patients’ electronic medical record is linked to these repository specimens, enabling translational studies on the virologic, immunologic, genetic and demographic determinants of HIV disease and associated comorbidities. The repository and allowable studies were reviewed and approved by the University of Washington Institutional Review Board (STUDY00001258). ACTU-2100 was a phase 2 open label study of mycophenolate mofetil for reduction of the HIV reservoir (NCT03262441) and was reviewed through the University of Washington Institutional Review Board (STUDY00002182). The Discordant Shedding Cohort study was approved by Institutional Review Boards in Lima, Peru (Hospital Nacional Dos de Mayo Comité de Etica) and Seattle, USA (Seattle Children’s Hospital Institutional Review Board; IRB #12035). The RAVEN cohort was approved by the University of California San Francisco Committee on Human Research (IRB #10–03244). RAVEN participants are enrolled and followed as part of the UCSF OPTIONS and SCOPE programs with specific consent for apheresis collections and testing for studies measuring the latent HIV reservoir. Participants in all studies were adults (> 18 years of age) and provided written, informed consent.

#### Cell lines and primary cells

##### Cell lines

HIV-1 infected J-Lat 8.4 T cells and HIV-uninfected Jurkat T cells, used to set up ddPCR assay controls, were cultured at 37°C, 5% CO_2_ in RPMI 1640 medium (with L-glutamine and HEPES) with 10% fetal bovine serum and 100 IU/mL of penicillin and 100ug/mL streptomycin. The J-Lat 8.4 T cells contain a single, full-length integrated copy of HIV-1. The J-Lat 8.4 T cells were obtained through the NIH AIDS Reagent Program, Division of AIDS, NIAID, NIH: J-Lat Full Length Clone (clone 8.4) from Dr. Eric Verdin Cat# 9847-427)^35^. The Jurkat T cell line was obtained through the American Type Culture Collection (ATCC): Jurkat Clone E6-1 from Dr. Arthur Weiss (Cat# TIB-152)^34^.

##### Primary cells

PBMC isolated from an HIV-negative human donor, used for *in vitro* subtype B HIV-1 infections, spiking linearized plasmid DNA with human DNA, and setting up ddPCR assay controls, were purchased from Bloodworks Northwest (https://www.bloodworksnw.org).

### Method details

#### Subtype B viral isolate infections of HIV-negative PBMCs

Five distinct subtype B viral isolates from the NIH International Panel of HIV-1 isolates^26^ were used to infect 1 × 10^6^ PBMC from an HIV-negative donor at an MOI of 0.02. Infected PBMCs were collected on day 19 of virus culture and DNA was extracted using the high-molecular weight method described below. For each viral isolate sample, we interrogated an average of 89,150 CD4^+^ T cells (range 14,900-163,900) across two replicates for both assay1 and assay2.

#### CD4^+^ T cell isolation

For all ddPCR assays except for the Discordant Shedding Cohort, we extracted gDNA from 1-2x10^6^ CD4^+^ T cells isolated by negative selection (StemCell EasySep™ Human CD4^+^ T Cell Isolation Kit). For the Discordant Shedding Cohort, PBMC pellets were dry frozen and CD4^+^ T cells could not be isolated; thus, gDNA from total PBMC was used.

#### DNA extraction and digestion

We tested several methods of extracting un-sheared gDNA with the goal of maximizing the amount of intact gDNA included in the reaction and minimizing the amount of sample manipulation and time required to complete the protocol. We chose a method that uses chaotropic salts to separate proteins and nucleic acids^28^ followed by a restriction enzyme digestion with *Bgl*I and a final ethanol precipitation of the digested genomic DNA^70^.

#### gDNA extraction for mucosal tissues

In addition to quantifying HIV in the periphery, it may also be valuable to measure HIV in mucosal tissues because the female genital tract is the main site of sexual transmission and the gastrointestinal tract is thought to be a major reservoir of latent virus. We combined our method for extracting high molecular weight gDNA from cells with the tissue lysis steps described in the protocol for QIAGEN’s QIAmp gDNA extraction kit (QIAamp DNA Micro Kit Cat#56304). Briefly, mucosal tissue biopsies trimmed to a maximum weight (after blotting to remove excess liquid) of 7mg are lysed in QIAGEN buffer ATL and proteinase K (QIAGEN 19131) overnight at 56°C until the tissue is dissolved. Once the tissue is dissolved, the protocol continues as for the extraction from cells, starting with the addition of 6M guanidine thiocyanate.

#### Droplet digital PCR

We ran two HIV assays and a reference assay (Tables S1–S3). Assay1 consisted of primer and probe mixtures for 3′pol, tat and env. Assay2 consisted of primer and probe mixtures for 5′pol, gag, and env (same env primers and probes for assay1 and assay2). The reference assay contained primer and probe mixtures for two regions of the *RPP30* gene and for the *TRD* gene, a region of the T cell receptor that is deleted during T cell development. The two regions of *RPP30* were separated by ∼11,000 nucleotides, approximately the same distance spanned by our HIV assays, and were used to estimate shearing. The *TRD* region is absent from T cells (“deltaD”) but present in all other cells, so allows quantification of the number of T cells interrogated^18^.

We followed the manufacturer’s protocols (Bio-Rad, ddPCR Supermix for Probes [No dUTP]) for master mix preparation, automatic droplet generation and thermal-cycling (Bio-Rad QX200 Droplet Digital PCR System) with the following exceptions: 1) we did not perform the restriction enzyme digestion as part of the thermal-cycling protocol because our chosen enzyme required a high-salt buffer that could interfere with downstream reactions. Instead, we extracted the nucleic acid out of the restriction digestion reaction by ethanol precipitation prior to thermal-cycling. 2) We increased the number of cycles of denaturation/annealing/extension to 60 cycles from 40 cycles. During preliminary tests, we found that increasing the number of cycles reduced the amount of “rain” (intermediate droplets) and consequently resulted in cleaner clusters and allowed better gating of the eight different populations, without increasing false positives^71^.

All template gDNA from a single participant was run on the same plate (i.e., samples from different time points were not split across plates). Each HIV assay (assay1 and assay2) was run in triplicate and the reference assay was run in duplicate. Two gating controls and two assay controls were also run on each plate. The gating controls were linearized plasmid DNA with spiked-in HIV-negative human PBMC DNA. There were two types of plasmid controls: plasmid control “P1” is a mixture of plasmids each containing the sequences from all possible combinations of our HIV targets, combined with HIV-negative human genomic DNA (Promega G3041). Plasmid control “P2” is a single plasmid containing all the targets, also with HIV-negative genomic DNA. The first control results in high numbers of droplets in all the possible populations and the second results in the majority of droplets in the “triple positive” population. The positive assay control was a mixture of 1 J-Lat 8.4 cells:1x10^3^ Jurkat cells. Negative assay controls were either Jurkat cells or HIV-negative PBMC.

#### Plasmid controls

We designed control plasmids using the pUCIDT_Amp vector backbone (Integrated DNA Technologies) with an insert containing all our primer/probe sequences and restriction enzyme cut sites. We then used traditional restriction enzyme cloning to create plasmids with primer sequences reflecting all possible combinations of genes from each of our two multiplex assays. To minimize the number of intermediate droplets (“rain”), we linearized the plasmid DNA with the restriction enzyme *Aat*II (New England BioLabs), then isolated the digested DNA by agarose gel electrophoresis and extracted the digested DNA using the QIAGEN QIAquick Gel Extraction Kit (Cat# 28704).

#### QVOA protocol

For the viral outgrowth assay performed on the UW-CFAR II cohort, CD4^+^ T cells were isolated by negative selection, serially diluted and plated with monocytes purified from an HIV-negative donor and cultured for three days with anti-CD3 OKT3 (Miltenyi, Cat. No. 130-093-387). At day 3 and 10, culture media was removed and CD8^+^ T cell-depleted allogeneic PHA-blasts from an HIV-negative donor, and IL-2, were added. Cultures were maintained for 28 days and virus outgrowth was measured by HIV p24 antigen. The infectious units per million CD4^+^ T cells (IUPM) were calculated using the maximum likelihood method^72^.

The RAVEN samples were measured by QVOA or differentiation QVOA as previously described^3^.

### Quantification and statistical analysis

#### Details of statistical tests (test type, etc.) are described in the text with results.

##### Cluster gating and data export

We auto-gated the P1 and P2 (plasmid mixed with HIV-negative human gDNA) controls in QuantaSoft Analysis Pro (version 1.0.596.0525) and then applied these gates to experimental samples. Cluster and well data were exported from QuantaSoft Analysis Pro and all subsequent data manipulation was carried out using R version 3.5.2. Plots were made in QuantaSoft Analysis Pro and Rstudio^73^, ^74^, ^75^, ^76^, ^77^, ^78^.

##### Concentration calculations

In QuantaSoft Analysis Pro, we designated all wells as “Amplitude Multiplex” and assigned the target name and corresponding FAM and HEX signals. For each sample and assay we merged the technical replicate wells by adding up the droplet counts from each technical replicate, excluding any wells that had fewer than 10,000 total droplets.

Next, we calculated DNA concentrations in copies per μL for each combination of targets that were detected in a given assay. QuantaSoft AP defines populations of droplets that are positive for the same target(s) as “clusters.” We used droplet counts from the cluster data exported from QuantaSoft AP and the following formula from the Bio-Rad applications guide to calculate the copies per μL of each cluster^79^.

Copies/μL = −ln (droplets not in cluster/total droplets in well)/droplet volume

Back-calculation of concentrations calculated by QuantaSoftAP for single targets (i.e., the total concentration of a target, regardless of whether it was in combination with another target) showed the assumed droplet volume to be 0.85nL, which has been reported in the literature^80^. We defined “total HIV” as the sum of the concentrations of each cluster of HIV targets.

##### Number of total cells, T cells and normalization of triple positive copies

To calculate the number of cells used in the HIV multiplex assay we multiplied the total copies per well for one of the targets in *RPP30* (“5′*RPP30*”) and deltaD by the DNA dilution factor (100), and divided the product by 2, because there are two copies of these targets in each diploid genome. The difference in number of copies of *RPP30* that we detected from the two *RPP30* assays was low (CV approximately 1%), so we chose to use the values from the 5′*RPP30*, the target closer to the 5′ end of *RPP30*, to calculate cells/μL. The difference between the two assays should be noted because a high CV may indicate a problem with the assay or data processing.

The deltaD region is present in all non-T cells and the *RPP30* target is present in all cells. Therefore, we calculated the number of T cells in each sample by subtracting the number of non-T cell genomes from the total genomes in the sample^18^. We normalized the number of triple positive copies to 1x10^6^ cells or T cells in the sample by multiplying 1x10^6^ by the ratio of triple positive copies to cells or T cells in the same sample.

##### Calculation of intact HIV proviral copies using shearing index correction

We used the results from our reference assay containing two targets in *RPP30* to calculate a DNA shearing index (DSI)^17^ for each sample (Figure 2F). The purpose of the DSI was to adjust for the fact that some provirus fragments did not contain all three targets because of mechanical shearing of the DNA (not due to mutation or deletion of the target regions). To adjust for shearing, we divided the number of triple positive droplets in each HIV assay (i.e., the number of potentially intact proviral copies) by the fraction of templates that were *not* sheared (1-DSI). Therefore, corrected triple positive copies (intact HIV copies) = observed triple positive copies / (1-DSI) (Figure 2F).

##### Calculation of defective HIV proviral copies

We calculated defective HIV proviral copies from this assay by subtracting intact HIV copies from total HIV copies. The calculation indirectly incorporates the shearing index correction, because the increased number of triple positive copies from the shearing correction is subtracted from the number of defective copies: Defective HIV equals Total HIV minus Intact HIV (D = T – I), whereby T = N_A_ + N_B_ + N_C_ + N_AB_ + N_BC_ + N_AC_ + N_ABC_, I = N_ABC_ / (1-DSI), and N_ABC_ = observed triple positive copies. ABC indicates that this count refers to triple positive copies only. Similarly, N_AB_, N_BC_ and N_AC_ refer to copies positive for two of the three targets, and N_A_, N_B_ and N_C_ refer to copies positive for only one of the three targets.

##### HIV reservoir half-life modeling

Using the longitudinal UW-CFAR-KINETICS data, we modeled a single-phase reservoir decay to estimate the half-life of defective and intact HIV DNA. Specifically, we assumed the HIV reservoir can be described with a simple exponential decay model^81^, $\partial_{t}L=\theta L$, where a value of the clearance slope θ and the initial reservoir size $L_{0}$ are estimated for each individual in each dataset using a population mixed effects modeling framework via the software Monolix^82^. Noise was assumed to be proportional to reservoir size, initial reservoir size was assumed to be log-normal, and clearance slope was assumed to be normal such that values could be non-negative (inclusive of no clearance). Half-life was then calculated as $t_{1/2}=-\ln\left(2\right)/\theta$, and converted to units of months.

##### Probit analysis

We used a probit analysis to calculate the Limit of Detection (LoD) for which 95% of true triple positive samples would be correctly identified as triple positive by the 5T-IPDA using IBM SPSS Version 26.

## Supplemental information

Document S1. Tables S1–S6 and Figures S1–S5

Document S2. Article plus supplemental information

## References

[1] Laird G.M., Eisele E.E., Rabi S.A., Lai J., Chioma S., Blankson J.N., Siliciano J.D., Siliciano R.F. (2013). Rapid quantification of the latent reservoir for HIV-1 using a viral outgrowth assay. PLoS Pathog..

[2] Sanyal A., Mailliard R.B., Rinaldo C.R., Ratner D., Ding M., Chen Y., Zerbato J.M., Giacobbi N.S., Venkatachari N.J., Patterson B.K. (2017). Novel assay reveals a large, inducible, replication-competent HIV-1 reservoir in resting CD4^+^ T cells. Nat. Med..

[3] Wonderlich E.R., Subramanian K., Cox B., Wiegand A., Lackman-Smith C., Bale M.J., Stone M., Hoh R., Kearney M.F., Maldarelli F. (2019). Effector memory differentiation increases detection of replication-competent HIV-l in resting CD4+ T cells from virally suppressed individuals. PLoS Pathog..

[4] Ho Y.C., Shan L., Hosmane N.N., Wang J., Laskey S.B., Rosenbloom D.I., Lai J., Blankson J.N., Siliciano J.D., Siliciano R.F. (2013). Replication-competent noninduced proviruses in the latent reservoir increase barrier to HIV-1 cure. Cell.

[5] Cohn L.B., Silva I.T., Oliveira T.Y., Rosales R.A., Parrish E.H., Learn G.H., Hahn B.H., Czartoski J.L., McElrath M.J., Lehmann C. (2015). HIV-1 integration landscape during latent and active infection. Cell.

[6] Bruner K.M., Murray A.J., Pollack R.A., Soliman M.G., Laskey S.B., Capoferri A.A., Lai J., Strain M.C., Lada S.M., Hoh R. (2016). Defective proviruses rapidly accumulate during acute HIV-1 infection. Nat. Med..

[7] Sengupta S., Siliciano R.F. (2018). Targeting the latent reservoir for HIV-1. Immunity.

[8] Bender A.M., Simonetti F.R., Kumar M.R., Fray E.J., Bruner K.M., Timmons A.E., Tai K.Y., Jenike K.M., Antar A.A.R., Liu P.T. (2019). The landscape of persistent viral genomes in ART-treated SIV, SHIV, and HIV-2 infections. Cell Host Microbe.

[9] Imamichi H., Dewar R.L., Adelsberger J.W., Rehm C.A., O’Doherty U., Paxinos E.E., Fauci A.S., Lane H.C. (2016). Defective HIV-1 proviruses produce novel protein-coding RNA species in HIV-infected patients on combination antiretroviral therapy. Proc. Natl. Acad. Sci. USA.

[10] Hiener B., Horsburgh B.A., Eden J.-S., Barton K., Lee E., Deeks S.G., Milush J., Chomont N., Fromentin R., Palmer S. (2017). Intact proviruses are unequally distributed in T cell subsets during ART. Top. Antivir. Med..

[11] Lee S.K., Kim C.J., Kim D.J., Kang J.H. (2015). Immune cells in the female reproductive tract. Immune Netw..

[12] Lee G.Q., Orlova-Fink N., Einkauf K., Chowdhury F.Z., Sun X., Harrington S., Kuo H.H., Hua S., Chen H.R., Ouyang Z. (2017). Clonal expansion of genome-intact HIV-1 in functionally polarized Th1 CD4+ T cells. J. Clin. Invest..

[13] Patro S.C., Brandt L.D., Bale M.J., Halvas E.K., Joseph K.W., Shao W., Wu X., Guo S., Murrell B., Wiegand A. (2019). Combined HIV-1 sequence and integration site analysis informs viral dynamics and allows reconstruction of replicating viral ancestors. Proc. Natl. Acad. Sci. USA.

[14] Hiener B., Horsburgh B.A., Eden J.S., Barton K., Schlub T.E., Lee E., von Stockenstrom S., Odevall L., Milush J.M., Liegler T. (2017). Identification of genetically intact HIV-1 proviruses in specific CD4^+^ T cells from effectively treated participants. Cell Rep..

[15] Salipante S.J., Jerome K.R. (2020). Digital PCR—an emerging technology with broad applications in microbiology. Clin. Chem..

[16] Strain M.C., Lada S.M., Luong T., Rought S.E., Gianella S., Terry V.H., Spina C.A., Woelk C.H., Richman D.D. (2013). Highly precise measurement of HIV DNA by droplet digital PCR. PLoS ONE.

[17] Bruner K.M., Wang Z., Simonetti F.R., Bender A.M., Kwon K.J., Sengupta S., Fray E.J., Beg S.A., Antar A.A.R., Jenike K.M. (2019). A quantitative approach for measuring the reservoir of latent HIV-1 proviruses. Nature.

[18] Zoutman W.H., Nell R.J., Versluis M., van Steenderen D., Lalai R.N., Out-Luiting J.J., de Lange M.J., Vermeer M.H., Langerak A.W., van der Velden P.A. (2017). Accurate quantification of T cells by measuring loss of germline T-cell receptor loci with generic single duplex droplet digital PCR assays. J. Mol. Diagn..

[19] Whale A.S., Huggett J.F., Tzonev S. (2016). Fundamentals of multiplexing with digital PCR. Biomol Detect. Quantif..

[20] Shao W., Shan J., Hu W.S., Halvas E.K., Mellors J.W., Coffin J.M., Kearney M.F. (2020). HIV Proviral Sequence Database: a new public database for near full-length HIV proviral sequences and their meta-analyses. AIDS Res. Hum. Retroviruses.

[21] Pagés H., Aboyoun P., Gentleman R., DebRoy S. (2019). Biostrings: efficient manipulation of biological strings. https://bioconductor.org/packages/release/bioc/html/Biostrings.html.

[22] Bembom O. (2018). seqLogo: sequence logos for DNA sequence alignments, R package version 1.48.0. https://bioconductor.org/packages/release/bioc/html/seqLogo.html.

[23] Ulrich J. (2019). TTR: Technical Trading Rules, R package version 0.23-6. https://cran.r-project.org/web/packages/TTR/index.html.

[24] Charif D., Lobry J.R., Bastolla U., Porto M., Roman E., Vendruscolo M. (2007). SeqinR 1.0-2: a contributed package to the R project for statistical computing devoted to biological sequences retrieval and analysis. Structural Approaches to Sequence Evolution: Molecules, Networks, Populations.

[25] Singh S.K., Koshkin A.A., Wengel J., Nielsen P. (1998). LNA (locked nucleic acids): synthesis and high-affinity nucleic acid recognition. Chem. Commun..

[26] Brown B.K., Darden J.M., Tovanabutra S., Oblander T., Frost J., Sanders-Buell E., de Souza M.S., Birx D.L., McCutchan F.E., Polonis V.R. (2005). Biologic and genetic characterization of a panel of 60 human immunodeficiency virus type 1 isolates, representing clades A, B, C, D, CRF01_AE, and CRF02_AG, for the development and assessment of candidate vaccines. J. Virol..

[27] Sambrook J., Russell D.W. (2006). Purification of nucleic acids by extraction with phenol:chloroform. CSH Protoc.

[28] Wiegand A., Spindler J., Hong F.F., Shao W., Cyktor J.C., Cillo A.R., Halvas E.K., Coffin J.M., Mellors J.W., Kearney M.F. (2017). Single-cell analysis of HIV-1 transcriptional activity reveals expression of proviruses in expanded clones during ART. Proc. Natl. Acad. Sci. USA.

[29] Morgan M., Anders S., Lawrence M., Aboyoun P., Pagès H., Gentleman R. (2009). ShortRead: a bioconductor package for input, quality assessment and exploration of high-throughput sequence data. Bioinformatics.

[30] Morgan M., Pagès H., Obenchain V., Hayden N. (2019). Rsamtools: binary alignment (BAM), FASTA, variant call (BCF), and tabix file import. http://bioconductor.org/packages/release/bioc/html/Rsamtools.html.

[31] The Bioconductor Dev Team (2015). BSgenome.Hsapiens.UCSC.hg38: full genome sequences for Homo sapiens (UCSC version hg38). https://bioconductor.org/packages/release/data/annotation/html/BSgenome.Hsapiens.UCSC.hg38.html.

[32] Pagés H. (2020). BSgenome: software infrastructure for efficient representation of full genomes and their SNPs, R package version 1.58.0. https://rdrr.io/bioc/BSgenome/.

[33] Kent W.J., Sugnet C.W., Furey T.S., Roskin K.M., Pringle T.H., Zahler A.M., Haussler D. (2002). The human genome browser at UCSC. Genome Res..

[34] Weiss A., Wiskocil R.L., Stobo J.D. (1984). The role of T3 surface molecules in the activation of human T cells: a two-stimulus requirement for IL 2 production reflects events occurring at a pre-translational level. J. Immunol..

[35] Jordan A., Bisgrove D., Verdin E. (2003). HIV reproducibly establishes a latent infection after acute infection of T cells in vitro. EMBO J..

[36] Kumar V., Vollbrecht T., Chernyshev M., Mohan S., Hanst B., Bavafa N., Lorenzo A., Kumar N., Ketteringham R., Eren K. (2019). Long-read amplicon denoising. Nucleic Acids Res..

[37] Edgar R.C. (2010). Search and clustering orders of magnitude faster than BLAST. Bioinformatics.

[38] Soria J., Bull M., Mitchell C., La Rosa A., Dross S., Kraft K., Coombs R., Ticona E., Frenkel L. (2012). Transmitted HIV resistance to first-line antiretroviral therapy in Lima, Peru. AIDS Res. Hum. Retroviruses.

[39] Abdel-Mohsen M., Richman D., Siliciano R.F., Nussenzweig M.C., Howell B.J., Martinez-Picado J., Chomont N., Bar K.J., Yu X.G., Lichterfeld M., BEAT-HIV Delaney Collaboratory to Cure HIV-1 infection (2020). Recommendations for measuring HIV reservoir size in cure-directed clinical trials. Nat. Med..

[40] Hosmane N.N., Kwon K.J., Bruner K.M., Capoferri A.A., Beg S., Rosenbloom D.I., Keele B.F., Ho Y.C., Siliciano J.D., Siliciano R.F. (2017). Proliferation of latently infected CD4^+^ T cells carrying replication-competent HIV-1: Potential role in latent reservoir dynamics. J. Exp. Med..

[41] Peluso M.J., Bacchetti P., Ritter K.D., Beg S., Lai J., Martin J.N., Hunt P.W., Henrich T.J., Siliciano J.D., Siliciano R.F. (2020). Differential decay of intact and defective proviral DNA in HIV-1-infected individuals on suppressive antiretroviral therapy. JCI Insight.

[42] Falcinelli S.D., Kilpatrick K.W., Read J., Murtagh R., Allard B., Ghofrani S., Kirchherr J., James K.S., Stuelke E., Baker C. (2020). Longitudinal dynamics of intact HIV proviral DNA and outgrowth virus frequencies in a cohort of ART-treated individuals. J. Infect. Dis..

[43] Siliciano J.D., Kajdas J., Finzi D., Quinn T.C., Chadwick K., Margolick J.B., Kovacs C., Gange S.J., Siliciano R.F. (2003). Long-term follow-up studies confirm the stability of the latent reservoir for HIV-1 in resting CD4+ T cells. Nat. Med..

[44] Crooks A.M., Bateson R., Cope A.B., Dahl N.P., Griggs M.K., Kuruc J.D., Gay C.L., Eron J.J., Margolis D.M., Bosch R.J., Archin N.M. (2015). Precise quantitation of the latent HIV-1 reservoir: Implications for eradication strategies. J. Infect. Dis..

[45] Finzi D., Blankson J., Siliciano J.D., Margolick J.B., Chadwick K., Pierson T., Smith K., Lisziewicz J., Lori F., Flexner C. (1999). Latent infection of CD4+ T cells provides a mechanism for lifelong persistence of HIV-1, even in patients on effective combination therapy. Nat. Med..

[46] Strain M.C., Günthard H.F., Havlir D.V., Ignacio C.C., Smith D.M., Leigh-Brown A.J., Macaranas T.R., Lam R.Y., Daly O.A., Fischer M. (2003). Heterogeneous clearance rates of long-lived lymphocytes infected with HIV: intrinsic stability predicts lifelong persistence. Proc. Natl. Acad. Sci. USA.

[47] Vibholm L.K., Lorenzi J.C.C., Pai J.A., Cohen Y.Z., Oliveira T.Y., Barton J.P., Garcia Noceda M., Lu C.L., Ablanedo-Terrazas Y., Del Rio Estrada P.M. (2019). Characterization of intact proviruses in blood and lymph node from HIV-infected individuals undergoing analytical treatment interruption. J. Virol..

[48] Banga R., Procopio F.A., Noto A., Pollakis G., Cavassini M., Ohmiti K., Corpataux J.M., de Leval L., Pantaleo G., Perreau M. (2016). PD-1(+) and follicular helper T cells are responsible for persistent HIV-1 transcription in treated aviremic individuals. Nat. Med..

[49] Chaillon A., Gianella S., Dellicour S., Rawlings S.A., Schlub T.E., De Oliveira M.F., Ignacio C., Porrachia M., Vrancken B., Smith D.M. (2020). HIV persists throughout deep tissues with repopulation from multiple anatomical sources. J. Clin. Invest..

[50] Chun T.W., Carruth L., Finzi D., Shen X., DiGiuseppe J.A., Taylor H., Hermankova M., Chadwick K., Margolick J., Quinn T.C. (1997). Quantification of latent tissue reservoirs and total body viral load in HIV-1 infection. Nature.

[51] Dufour C., Gantner P., Fromentin R., Chomont N. (2020). The multifaceted nature of HIV latency. J. Clin. Invest..

[52] Estes J.D., Kityo C., Ssali F., Swainson L., Makamdop K.N., Del Prete G.Q., Deeks S.G., Luciw P.A., Chipman J.G., Beilman G.J. (2017). Defining total-body AIDS-virus burden with implications for curative strategies. Nat. Med..

[53] Belmonte L., Olmos M., Fanin A., Parodi C., Baré P., Concetti H., Pérez H., de Bracco M.M., Cahn P. (2007). The intestinal mucosa as a reservoir of HIV-1 infection after successful HAART. AIDS.

[54] Chun T.W., Nickle D.C., Justement J.S., Meyers J.H., Roby G., Hallahan C.W., Kottilil S., Moir S., Mican J.M., Mullins J.I. (2008). Persistence of HIV in gut-associated lymphoid tissue despite long-term antiretroviral therapy. J. Infect. Dis..

[55] Yukl S.A., Gianella S., Sinclair E., Epling L., Li Q., Duan L., Choi A.L., Girling V., Ho T., Li P. (2010). Differences in HIV burden and immune activation within the gut of HIV-positive patients receiving suppressive antiretroviral therapy. J. Infect. Dis..

[56] Gornalusse G.G., Valdez R., Fenkart G., Vojtech L., Fleming L.M., Pandey U., Hughes S.M., Levy C.N., Dela Cruz E.J., Calienes F.L. (2020). Mechanisms of endogenous HIV-1 reactivation by endocervical epithelial cells. J. Virol..

[57] Bull M., Mitchell C., Soria J., Styrchak S., Williams C., Dragavon J., Ryan K.J., Acosta E., Onchiri F., Coombs R.W. (2020). Genital shedding of human immunodeficiency virus type-1 (HIV) when antiretroviral therapy suppresses HIV replication in the plasma. J. Infect. Dis..

[58] Bull M.E., Learn G.H., McElhone S., Hitti J., Lockhart D., Holte S., Dragavon J., Coombs R.W., Mullins J.I., Frenkel L.M. (2009). Monotypic human immunodeficiency virus type 1 genotypes across the uterine cervix and in blood suggest proliferation of cells with provirus. J. Virol..

[59] Hughes S.M., Ferre A.L., Yandura S.E., Shetler C., Baker C.A.R., Calienes F., Levy C.N., Astronomo R.D., Shu Z., Lentz G.M. (2018). Cryopreservation of human mucosal tissues. PLoS ONE.

[60] Hughes S.M., Shu Z., Levy C.N., Ferre A.L., Hartig H., Fang C., Lentz G., Fialkow M., Kirby A.C., Adams Waldorf K.M. (2016). Cryopreservation of human mucosal leukocytes. PLoS ONE.

[61] Kumar A., Abbas W., Herbein G. (2014). HIV-1 latency in monocytes/macrophages. Viruses.

[62] Gaebler C., Falcinelli S.D., Stoffel E., Read J., Murtagh R., Oliveira T.Y., Ramos V., Lorenzi J.C.C., Kirchherr J., James K.S. (2021). Sequence evaluation and comparative analysis of novel assays for intact proviral HIV-1 DNA. J. Virol..

[63] Kinloch N.N., Ren Y., Conce Alberto W.D., Dong W., Khadka P., Huang S.H., Mota T.M., Wilson A., Shahid A., Kirkby D. (2021). HIV-1 diversity considerations in the application of the Intact Proviral DNA Assay (IPDA). Nat. Commun..

[64] Wagner T.A., McLaughlin S., Garg K., Cheung C.Y., Larsen B.B., Styrchak S., Huang H.C., Edlefsen P.T., Mullins J.I., Frenkel L.M. (2014). HIV latency. Proliferation of cells with HIV integrated into cancer genes contributes to persistent infection. Science.

[65] Maldarelli F., Wu X., Su L., Simonetti F.R., Shao W., Hill S., Spindler J., Ferris A.L., Mellors J.W., Kearney M.F. (2014). HIV latency. Specific HIV integration sites are linked to clonal expansion and persistence of infected cells. Science.

[66] Lenasi T., Contreras X., Peterlin B.M. (2008). Transcriptional interference antagonizes proviral gene expression to promote HIV latency. Cell Host Microbe.

[67] Einkauf K.B., Lee G.Q., Gao C., Sharaf R., Sun X., Hua S., Chen S.M., Jiang C., Lian X., Chowdhury F.Z. (2019). Intact HIV-1 proviruses accumulate at distinct chromosomal positions during prolonged antiretroviral therapy. J. Clin. Invest..

[68] Jiang C., Lian X., Gao C., Sun X., Einkauf K.B., Chevalier J.M., Chen S.M.Y., Hua S., Rhee B., Chang K. (2020). Distinct viral reservoirs in individuals with spontaneous control of HIV-1. Nature.

[69] Taylor B.S., Sobieszczyk M.E., McCutchan F.E., Hammer S.M. (2008). The challenge of HIV-1 subtype diversity. N. Engl. J. Med..

[70] Green M.R., Sambrook J. (2016). Precipitation of DNA with ethanol. Cold Spring Harb. Protoc..

[71] Witte A.K., Mester P., Fister S., Witte M., Schoder D., Rossmanith P. (2016). A systematic investigation of parameters influencing droplet rain in the Listeria monocytogenes prfA assay - reduction of ambiguous results in ddPCR. PLoS ONE.

[72] Rosenbloom D.I.S., Elliott O., Hill A.L., Henrich T.J., Siliciano J.M., Siliciano R.F. (2015). Designing and interpreting limiting dilution assays: general principles and applications to the latent reservoir for human immunodeficiency virus-1. Open Forum Infect. Dis..

[73] Attali D. (2019). ddpcr: analysis and visualization of droplet digital PCR in R and on the Web, R package version 1.11. https://cran.microsoft.com/snapshot/2019-12-24/web/packages/ddpcr/index.html.

[74] Wickham H. (2017). tidyverse: easily install and load the ‘Tidyverse’. https://cran.r-project.org/web/packages/tidyverse/index.html.

[75] RStudio (2015). RStudio: integrated development for R. https://rstudio.com/.

[76] R Development Core Team (2018). R: A language and environment for statistical computing.

[77] Hughes S.M. (2016). plater: read, tidy, and display data from microtiter plates. J. Open Source Softw..

[78] Grolemund G., Wickham H. (2011). Dates and times made easy with lubridate. J. Stat. Softw..

[79] Bio-Rad (2014). Droplet Digital PCR Applications Guide. http://www.bio-rad.com/webroot/web/pdf/lsr/literature/Bulletin_6407.pdf.

[80] Corbisier P., Pinheiro L., Mazoua S., Kortekaas A.M., Chung P.Y., Gerganova T., Roebben G., Emons H., Emslie K. (2015). DNA copy number concentration measured by digital and droplet digital quantitative PCR using certified reference materials. Anal. Bioanal. Chem..

[81] Reeves D.B., Duke E.R., Hughes S.M., Prlic M., Hladik F., Schiffer J.T. (2017). Anti-proliferative therapy for HIV cure: a compound interest approach. Sci. Rep..

[82] Kuhn E., Lavielle M. (2005). Maximum likelihood estimation in nonlinear mixed effects models. Comput. Stat. Data Anal..

