# Supplementary material for: A highly multiplexed droplet digital PCR assay to measure the intact HIV-1 proviral reservoir
Source: Cell Rep Med. 2021 Apr 12;2(4):100243. doi: 10.1016/j.xcrm.2021.100243 (PMC8080125; doi:10.1016/j.xcrm.2021.100243)
Supplement: Document S1. Tables S1–S6 and Figures S1–S5 [file mmc1.pdf]

Cell Reports Medicine, Volume *2*

Supplemental information

A highly multiplexed droplet digital PCR assay

to measure the intact HIV-1 proviral reservoir

Claire N. Levy, Sean M. Hughes, Pavitra Roychoudhury, Daniel B. Reeves, Chelsea Amstuz, Haiying Zhu, Meei-Li Huang, Yulun Wei, Marta E. Bull, Noah A.J. Cassidy, Jan McClure, Lisa M. Frenkel, Mars Stone, Sonia Bakkour, Elizabeth R. Wonderlich, Michael P. Busch, Steven G. Deeks, Joshua T. Schiffer, Robert W. Coombs, Dara A. Lehman, Keith R. Jerome, and Florian Hladik

Supplemental information

Supplemental tables

Multiplex assay	Primer/ probe	Dye, Quencher	Sequence 5' to 3'	NC_001802 coordinates downloaded 27June19
assay1	3'pol F		AGAGATCCACTTTGGAAAGGACCAGCAAA	4466-4494
	3'pol probe	FAM, BHQ	TGGAAAGGTGAAGG	4502-4515
	3'pol R		CACTACTTTTATGTCACTATTATCTTGTATTACTACTGC	4517-4555
	tat F		TTTGTTTCATAACAAAAGGCTTAGGCATCTCC	5483-5514
	tat probe	FAM, BHQ	ATGGCAGGAAGAAG	5516-5529
	tat R		TGAGGAGCTCTTCGTCGCTGTCTCC	5531-5555
assay2	5' pol F		TCCTTTAGCTTCCCTCAGATCACTCT	1787-1812
	5' pol probe	FAM, BHQ	TTGGCAACGACC	1813-1824
	5' pol R		TACTGTATCATCTGCTCCTGTATCTAATAGAGCTTC	1859-1894
	gag F		GACTAGCGGAGGCTAGAAGGAGAGA	310-334
	gag probe	HEX, BHQ	ATGGGTGCGAGA	336-347
	gag R		CTAATTCTCCCCCGCTTAATAYTGACG	345-375
assay1 & assay2	env F		TGTTCCTTGGGTTCTTGGGAGCAGCAGG	7323-7350
	env probe	HEX, BHQ	AGCACTATGGG	7352-7362
	env R		GCACTATACCAGACAATAATTGTCTGGCCTGTACC	7384-7418

Table S1. HIV-1 multiplex primers and probes. Related to Figures 1 and 2.

Primer/ probe	Multiplex assay	Dye, Quencher	Sequence 5' to 3'	GenBank Gene ID 6964 (TRD) coordinates downloaded 27June19
deltaD F	RPP30_deltaD		GCTGGCTGTAATGGGAATGT	18144-18163
deltaD probe		FAM, BHQ	TGTGAAGATGTCTGTAGCCATCTTAT	18171-18196
deltaD R			TAATGGCTTGATAAAGATAAGTGATCAT	18201-18228

Table S2. Primers and probes for the deltaD target in *TRD*. Related to Figures 1 and 2.

Primer/probe	Multiplex assay	Dye, Quencher	Sequence 5' to 3'	GenBank Gene ID 10556 (*RPP30*) coordinates downloaded 24June19
5'*RPP30* F	RPP30_deltaD		GCATATCAGGGTACAGCATAGG	16599-16620
5'RPP30 probe		FAM, ZEN/IBFQ	TCTGCTCGTTGTTAGTCACCAGCT	16635-16658
5'*RPP30* R			CTTCCCTCACGGCATATACTTC	16678-16699
3'*RPP30* F			GCTGTGTTTGCTCTCTTGATTT	28266-28287
3'*RPP30* probe		HEX, ZEN/IBFQ	AATGTCTGTGACTGGGTTCTGGCT	28322-28345
3'*RPP30* R			GGTCTGTCCATGGCATCTTAT	28348-28368

Table S3. Primers and probes for the two targets in *RPP30*. Related to Figures 1 and 2.

Sample	Number of wells	Triple positive droplets		Triple positive detection rate
		assay1	assay2	
Promega HIV- PBMC gDNA	90	0	0	0/90
Lab extracted HIV- CD4+ gDNA	29	1	0	0/29
Lab extracted Jurkat gDNA	30	0	0	0/30

Table S4. Specificity (true negative rate) test results. Related to Figure 1.

J-Lat8.4: 1E6 Jurkat	Number of wells	Triple positive droplets		Triple positive detection rate
		assay1	assay2	
5000	14	14	14	14/14
2500	14	14	14	14/14
1000	14	14	14	14/14
500	14	14	14	14/14
100	14	13	14	13/14
50	13	13	12	12/13
5	14	10	4	4/14
1	14	5	1	1/14
0	14	0	0	0/14

Table S5. Limit of blank determination by dilution series. ~245,000 cells were used in each test. Related to Figure 1.

Cohort	Participant ID	Birth Year	Birth Sex	Location	ART at sampling	Time on ART	Plasma Viral Load copies/mL	Genital Viral Load copies/mL	CD4 count
UW-CFAR_KINETICS	1020	1963	M	Seattle, WA, USA	See Fig. S3	16 years	See Fig. S4	NA	See Fig. S5
	1028	1958	M			10 years			
	1029	1961	M			6 years			
	1031	1961	M			12 years			
	1036	1961	M			9 years			
	1068	1945	M			13 years			
	1097	1967	M			7 years			
	1186	1969	M			8 years			
	1231	1958	M			13 years			
	1268	1939	M			15 years			
	1269	1961	M			6 years			
	1308	1956	F			8 years			
	1320	1955	M			7 years			
	1332	1948	M			8 years			
	1370	1960	M			9 years			
	1377	1960	M			9 years			
	1401	1969	M			7 years			
	1444	1949	M			7 years			
	1819	1955	M			9 years			
	1825	1957	M			6 years			
Discordant Shedding	28	Not available	F	Lima, Peru	ddI/LPV / RTV	1 year	5292.00	171	Not available
	90-2		F		3TC/NVP/d4T	3 months	1000000.00	15,597	Not available
	90-4		F		EFV/3TC/d4T/NVP	9 months	1570.00	<30	Not available
	93		F		EFV/3TC/ZDV	6 months	4250.00	<30	694
	97		F		EFV/3TC/d4T	6 months	69.00	130	154
	101		F		EFV/3TC/ZDV	12 months	1694.00	<30	74
ACTU-2100	8628	1958	M	Seattle, WA, USA	EVG/COBI/FTC/TDF	19 years	visit 1: <7; visit 2: <7	NA	573
	9252	1963	M		DTG/FTC/TAF	16 years	visit 1: <7; visit 2: <7	NA	492
	9282	1992	M		DTG/RPV	5 years	visit 1: Undetectable; visit 2: Undetectable	NA	606
UW-CFAR_QVOA	AG	Not available	Not available	Seattle, WA, USA	Not available	Not available	<40	NA	Not available
	BK	1968	Not available		Not available	Not available	121		Not available
	BF	1978	M		EVG/COBI/FTC/TAF	4.5 years	<40		247
	BP	1964	M		DTG, TDF/FTC	15.1 years	<40		638
	BS	Not available	M		EVG/COBI/FTC/TAF	5.7 years	<40		554
	BT	1966	M		Not available	Not available	<40		399
	BU	1960	M		Not available	Not available	<40		399
	BW	1973	M		RTV, DRV, TDF/FTC	16.4 years	<40		616
	BX	1972	M		ATV, RTV, FTC/TDF	8.0 years	<40		545
	BY	1964	M		ATV, FTC, TDF	20.2 years	<40		635
	AA	1956	M		ABC/DTG/3TC	20.4 years	<40		580
	AB	1964	M		Not available	NA	<40		720
	AC	1966	M		Not available	NA	<40		500
	AF	1982	M		EVG/COBI/FTC/TAF	3 years	<40		1283
RAVEN	1602-R_2015-08-12	Not available	M	San Francisco, CA, USA	Not available	3.5 years	1.30	NA	626
	1602-R_2016-02-24		M		Not available	4.1 years	1.52		546
	2078-R_2015-05-06		M		Not available	15.3 years	0.80		589
	2168-R_2016-02-09		M		Not available	14.2 years	0.38		413
	2168-R_2016-08-15		M		Not available	14.7 years	0.07		318
	2274-R_2016-08-17		M		NVP, TDF/FTC	13.1 years	0.07		344
	2274-R_2018-03-27		M		Not available	Not available	Not available		Not available
	2511-R_2016-06-01		M		EFV/TDF/FTC, RAL	10.4 years	0.15		397
	2609-R_2015-11-09		M		Not available	1.5 years	1.52		899
	2672-R_2016-09-06		M		Not available	1.2 years	11.78		347

Table S6. Demographics of the UW-CFAR_KINETICS, UW-CFAR_QVOA, ACTU-2100, Discordant Shedding Cohort, and RAVEN participants. 3TC, lamivudine; ABC, abacavir; ATV, atazanavir; COBI, cobicistat; d4T, stavudine, ddI, didanosine; DRV, darunavir; DTG, dolutegravir; EFV, efavirenz; EVG, elvitegravir; NVP, nevirapine; RAL, raltegravir; RTV, ritonavir; ZDV, zidovudine. Participant 90 provided samples from two visits, their ID is appended with the visit number. NA, not applicable. Related to Figures 4, 5 and 6.

Supplemental Figures

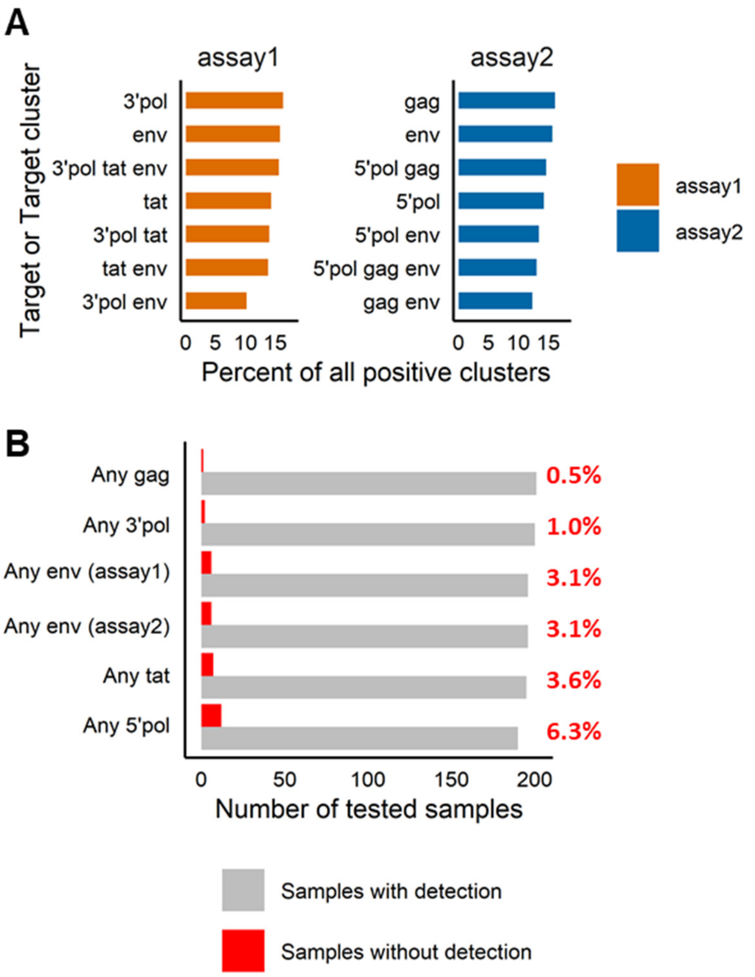

Figure S1. Target detection frequencies. (A) Detection frequencies of individual targets and target combinations in the two ddPCR assays. Percentages were calculated as 100 x events in specified cluster/events in all positive clusters. The *env* probe is the same sequence for both assays, so is measured twice for each sample. N = 201 for assay1 and N = 200 for assay2. Samples from $CD4^+$ gDNA from the UW-CFAR_KINETICS (n = 157), RAVEN (n = 17 for assay1, n = 16 for assay2) and UW-CFAR_QVOA (n = 27) cohorts. Note that the true frequencies of all clusters are impacted by the level of DNA shearing. **(B)** Detection frequencies of each of the five targets in any combination. Values to the right of each bar indicate the percentage of samples in which the target was not detected (N = 202; one sample was entirely negative and was included in the analysis in B but could not be included in A). Related to Figure 4.

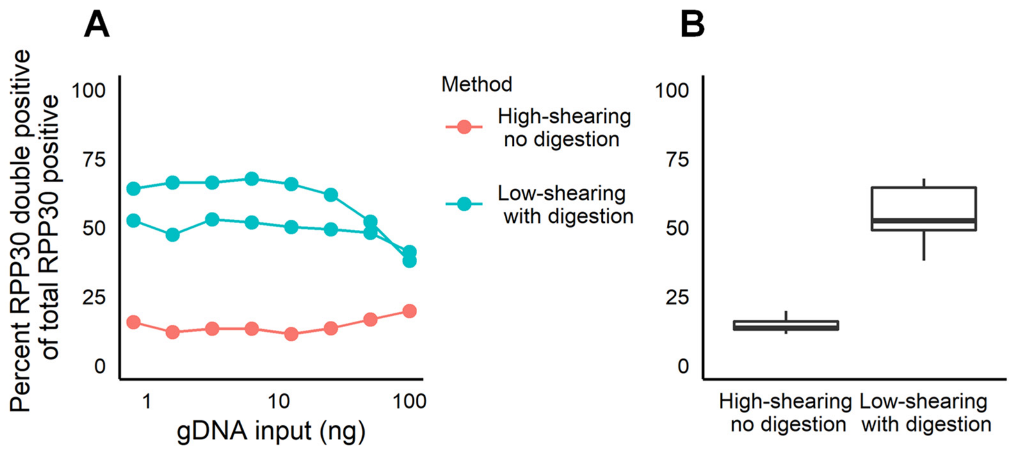

Figure S2. Comparison of shearing resulting from two extraction methods. We assayed two targets approximately 11kbp apart in the *RPP30* gene to assess shearing of the genomic DNA template. A higher percentage of *RPP30* double positive targets indicates less DNA shearing; more droplets contain DNA fragments containing both targets. **(A)** For clinical samples, a maximum of 10ng of gDNA is used per ddPCR replicate. For this demonstrative experiment, we used 8 different amounts of template from 1 to 100ng of gDNA template from ectocervical biopsies. If the gDNA input is increased to 100ng, the results from the two methods begin to converge because double positive droplets occur by chance due to the larger amount of DNA present. We extracted one replicate using the high-shearing method and two replicates with the low-shearing method. **(B)** A boxplot of showing the same data as in (A) but with the results of all 8 different amounts of DNA summarized in box and whisker plots. Related to Figure 3.

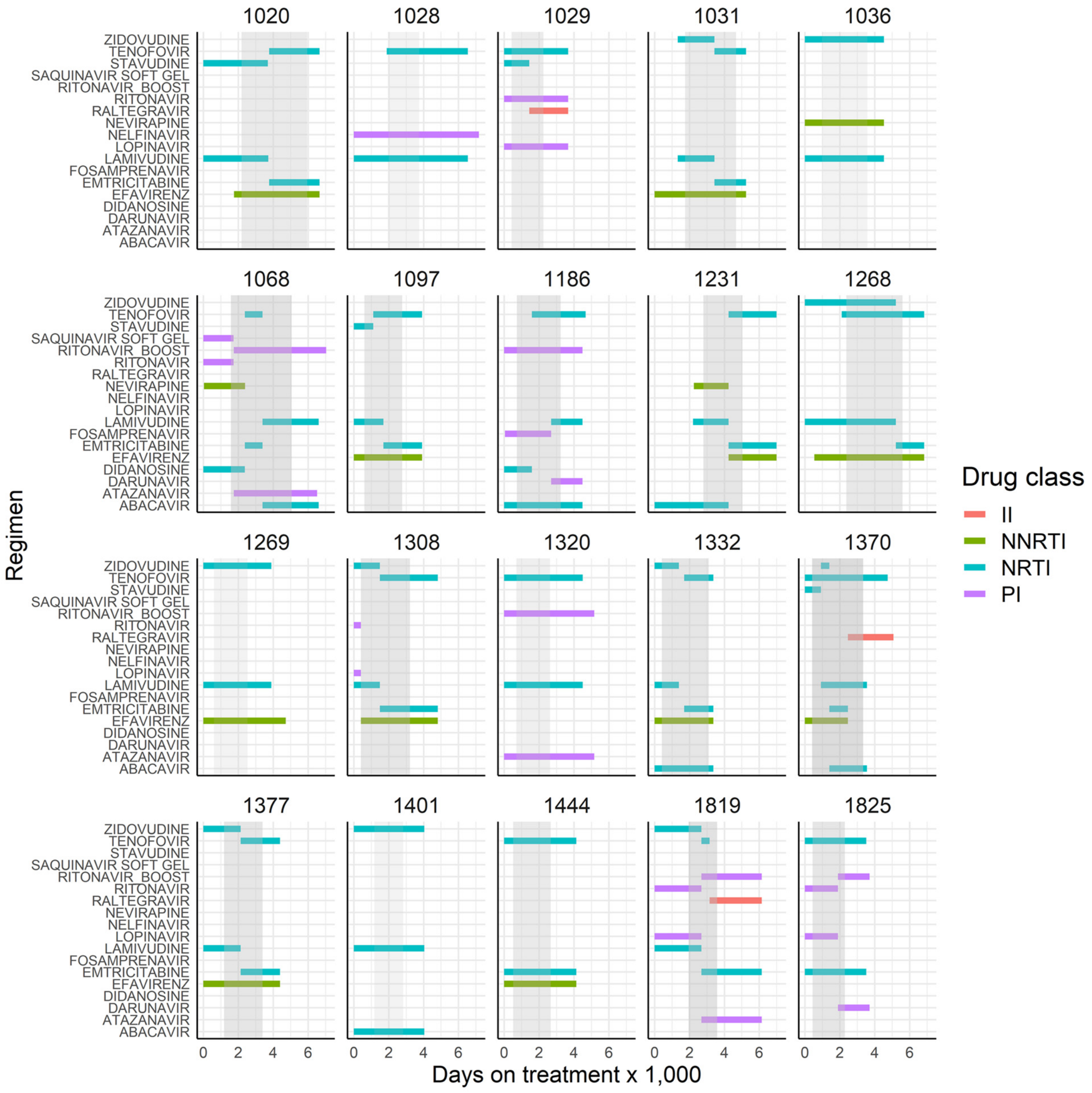

Figure S3. ART regimens over time for the 20 UW-CFAR_KINETICS cohort participants. II: integrase inhibitor; NNRTI: non-nucleoside reverse transcriptase inhibitor, NRTI: nucleoside reverse transcriptase inhibitor; PI: protease inhibitor. Gray shaded time span is when samples used in the present study were collected. Related to Figure 5.

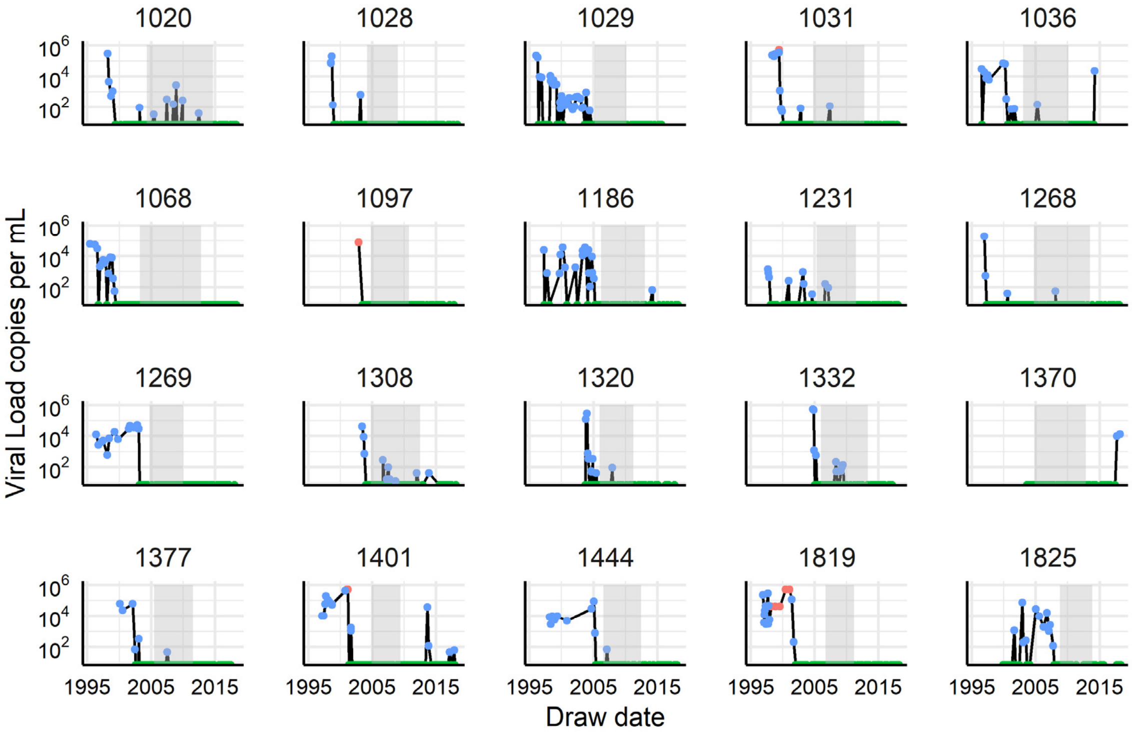

Figure S4. Viral loads over time for the 20 UW-CFAR_KINETICS cohort participants. Blue: result in detectable range; red: result above limit of quantitation; green: result undetectable/below limit of quantitation. Gray shaded time span is when samples used in the present study were collected. Viral load assay methods changed over time; detectability is relative to the particular assay used and may not be identical from point to point. Related to Figure 5.

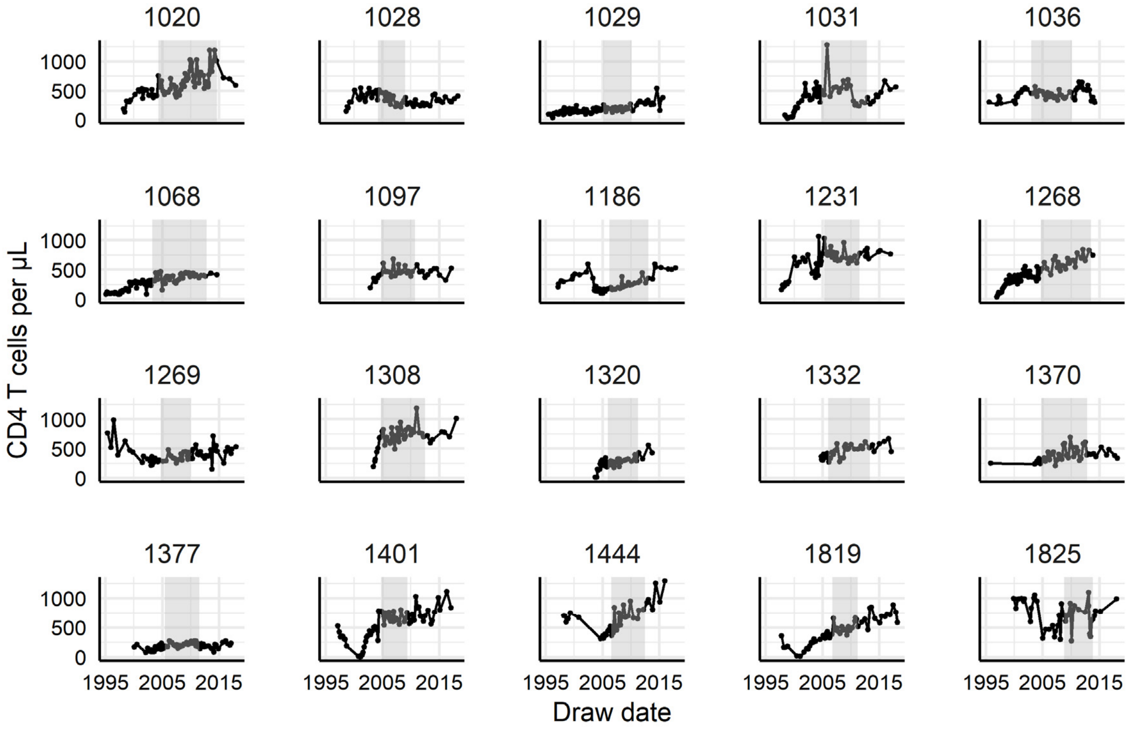

Figure S5. CD4⁺ T cell counts over time for the 20 UW-CFAR_KINETICS cohort participants. Gray shaded time span is when samples used in the present study were collected. Related to Figure 5.
